# Magnetically nanorized seaweed residue for the adsorption of methylene blue in aqueous solutions†

**DOI:** 10.1039/d4ra04416a

**Published:** 2024-07-29

**Authors:** Xinyi Yang, Jingjing Liu, Xuejin Huang, Hemin Cui, Ligang Wei, Guolin Shao, Xu Fu, Na Liu, Qingda An, Shangru Zhai

**Affiliations:** a School of Light Industry and Chemical Engineering, Dalian Polytechnic University Dalian 116034 China weilg@dlpu.edu.cn fuxumail@163.com +86 0411 86323726; b Liaoning Key Lab of Lignocellulose Chemistry and BioMaterials, Liaoning Collaborative Innovation Center for Lignocellulosic Biorefinery, Dalian Polytechnic University, Dalian Polytechnic University Dalian 116034 China; c Dalian Zhonghuida Scientific Instrument Co. Ltd Dalian 116023 China

## Abstract

The cost-effective and green separation of dye pollutants from wastewater is of great importance in environmental remediation. Industrial seaweed residue (SR), as a low-cost cellulose source, was used to produce carboxylated nanorized-SR (NSR) *via* oxalic acid (OA)–water pretreatments followed by ultrasonic disintegration. Fourier transform infrared spectroscopy, X-ray polycrystalline diffraction, nitrogen isotherms, scanning electron microscopy, transmission electron microscopy, vibrating sample magnetometry, X-ray photoelectron spectrometry, particle charge detection, zeta potential and retro titration experiments were utilized to explore the physiochemical properties of samples. The NSRs with carboxyl content of 4.58–6.73 mmol g^−1^ were prepared using 10–60% OA–water pretreatment. In the case of 20% OA–water pretreatment, the highest NSR yield (73.9%) and nanocellulose content (80.2%) were obtained. Through self-assembly induced by the electrostatic interaction, magnetic NSR composite adsorbents (MNSRs) were prepared with the combination of NSR and Fe_3_O_4_ nanoparticles (NPs). The carboxylated NSR with negative charge demonstrated good affinity for Fe_3_O_4_ NPs. The Fe_3_O_4_ NPs were perfectly microencapsulated with the NSR when the NSR/Fe_3_O_4_ mass ratio was higher than 1/1. The adsorption properties of the MNSR for methylene blue (MB) removal from aqueous solution were investigated. The adsorbent with NSR/Fe_3_O_4_ mass ratio of 1/1 (MNSR1/1) exhibited optimum performance in terms of the magnetic properties and adsorption capacity. The MNSR1/1 showed high adsorption ability in a pH ≥7 environment. According to the Langmuir fitting, the maximum adsorption capacity of MNSR1/1 for MB reached 184.25 mg g^−1^. The adsorption of MB complies with the pseudo-second-order kinetic model. MNSR1/1 still maintained good adsorption properties after the fifth cycle of adsorption–desorption. MNSR1/1 could selectively adsorb cationic dye (*i.e.*, MB and methyl violet) from wastewater, with hydrogen bonding and electrostatic interaction as the main force.

## Abstract 2

Carboxylated nanorized-seaweed residue (NSR) produce *via* OA–water pretreatments. A magnetic NSR–Fe_3_O_4_ nanocomposite was synthesized by electrostatic interaction-induced aggregation for dye adsorption.

## Graphical abstract

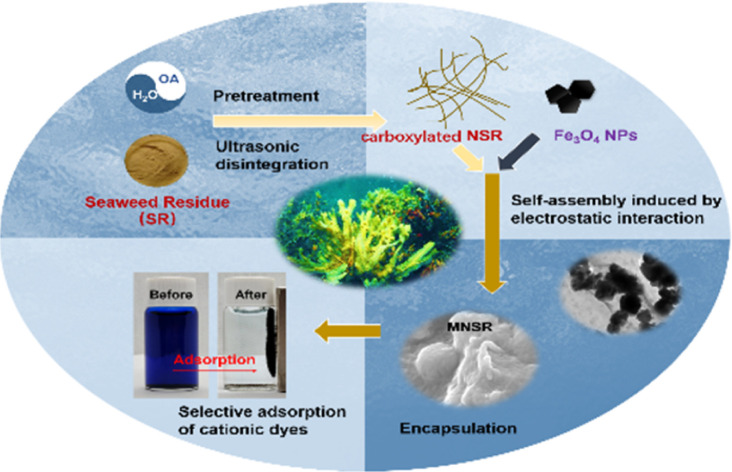

## 1. Introduction

With the rapid development of modern industries, water sources are currently faced with serious environmental problems. A large amount of pollutants, such as organic chemicals and heavy metal ions, exist in industrial wastewater.^1^ The dyes widely used in textiles, paper-making, leather, coating, printing, and dyeing industries are some of the main organic contaminants that need to be dealt with because they are threatening people's health.^2,3^ A series of methods have been proposed and employed to treat wastewater, including adsorption,^4^ membrane separation,^5^ chemical degradation,^6^ and electric/photocatalytic degradation.^7^ The adsorption processes are extensively used because of their advantages of simple operation, high efficiency, low cost, absence of secondary pollution, and zero alterations in the dye's chemical structure.^8^

At present, the use of biomass in water treatment is attracting interest. Cellulose, the most abundant biopolymer on earth, is a remarkable raw biomass material offering wide availability, sustainability, good processability, and the possibility of surface modification.^9^ Nanostructured adsorbent materials with high specific area offer higher adsorption capacities and better binding affinities than precursors in the macroscale.^10^ The emergence of nanocellulose (NC) as a novel material with a wide range of potential applications has propelled its application as a new generation of bio-based adsorbents. Cellulose nanocrystals (CNCs), cellulose nanofibrils (CNFs), and bacterial cellulose are the main families of NC, but they differ in their production mode and morphologies.^11^

He *et al.*^12^ prepared CNCs with a high specific area (248 m^2^ g^−1^) through the hydrolysis of microcrystalline cellulose in 1 M ammonium persulfate. The adsorption capacity of the prepared CNCs for methylene blue (MB), a kind of cationic dye, reached 101 mg g^−1^. The Langmuir isotherm model is correlated with the data of MB adsorption on the CNCs, indicating the homogeneous nature of the CNC surface. Chan *et al.*^13^ prepared CNFs from a kenaf core *via* acid–chlorite pretreatment, followed by disintegration using a high-speed blender. The prepared CNFs were used for MB adsorption, and the maximum adsorption capacity was 122.2 mg g^−1^ under 20 °C and pH 9. Rapid adsorption equilibrium was achieved within a contact time of 1 min. The NC-based adsorbents demonstrated improved adsorption capacities toward dyes. However, the limitations of NC must be addressed before it is considered for real water treatment.^14^

One of the key challenges for the future growth and integration of NC as a new class of sustainable adsorbent for dye adsorption is adsorbent recovery.^15^ The adsorbents in powder or particle forms have to be recovered and reused in practical applications. A convenient collection method is required for the adsorbents. Magnetically modified adsorbents can be selectively, rapidly, and easily separated from a specific environment (*i.e.*, wastewater) by using permanent magnets or magnetic separators.^16–18^ Recently, various magnetic lignocellulose composites have been employed for dye removal from aqueous solutions, such as microcrystalline cellulose, waste flax seeds, tea leaves, corn straw, bagasse, and sawdust.^19^ Zarei *et al.*^20^ prepared Fe_3_O_4_–CNC composites through the sol–gel method but investigated the uptake capacities of Hg(ii) ions instead of dye adsorption. The abovementioned works allowed us to infer the applicability and availability of magnetic NC composites for dye adsorption, although the relevant information is limited.

Another key challenge for the development and applications of NC-based adsorbents is cost efficiency.^15^ The preparation of NC from low-cost wastes containing cellulose is an effective way to reduce cost and avoid high energy consumption. In China, a large number of seaweed residues (SRs) rich in cellulose (up to 50%) are produced after alginate extraction.^21^ SRs are commonly used as animal feeds or fertilizers. The price of SR is lower than that of microcrystalline cellulose or pulp. As the industrial waste of seaweed processing, the supply of SR is more consistent and abundant than that of woody and agricultural biomass. These above issues allow SR to be particularly suitable for the preparation of NC. The preparation of high value-added nanorized SR (NSR) is rarely reported in the literature,^21^ let alone the application of NSR in dye removal.

In this study, SR was nanorized through the pretreatment of oxalic acid (OA)–water, followed by ultrasonic disintegration. The obtained NSR could contain NC in the nanoscale and a small amount of undisintegrated SR blocks with size of 1 μm and above. Compared with concentrated mineral acid, OA is environmentally benign and can be easily recovered simply through commercially proven crystalliztaion processes; moreover, the strict equipment requirement for corrosion prevention is not a major concern in OA–water utilization.^22^ These issues mean that OA–water reagent can be applied as a green reaction for functional NC production. When using OA, the hydrolyzed cellulosic materials can be functionalized with carboxyl groups through Fisher–Speier esterification of one carboxyl group (Scheme S1 in ESI†), which improves the compatibility of materials and the binding with dye or metal ions.^22^

The effects of the OA content on the adsorption capacity of NSR for cationic dye MB were evaluated. MB is widely used in the textile industry.^23^ It is frequently studied in the research area as a model adsorbate of organic contaminants in aqueous solutions because of its high affinity for binding to solids.^23–25^ In this study, a novel magnetic nanocomposite, NSR–Fe_3_O_4_ (MNSR), was successfully synthesized by simple electrostatic interaction-induced aggregation with ultrasonic assistance. The resulting magnetic nanocomposite was then utilized for MB adsorption from an aquatic environment. To our knowledge, this is the first time to use MNSR nanocomposite for removing environmental contaminants. The structure, morphology, and properties of MNSR composites were evaluated by a series of measurements. The adsorption characteristics of MNSR for MB dye were investigated in terms of kinetics and thermodynamics, and possible adsorption mechanisms were also proposed.

## 2. Experimental

### 2.1 Materials

Magnetic Fe_3_O_4_ nanoparticles (NPs; 99.9% metal basis) were provided by Beesley New Materials (Su Zhou) Co., Ltd (China). SR was supplied by Qingdao Hisea Imp. & Exp. Co., Ltd (China). According to the analytical procedures (GB/T 5009.10-2003, GB/T 5009.4-2016, GB/T 14772-2008, GB/T 5750.4-2006, Chinese standard method), the contents of cellulose, ash, lipid, and water-soluble compounds were 65.0 ± 3.2%, 24.7 ± 2.8%, 3.9 ± 0.5%, and 6.6 ± 0.9% (weight percentage; used in subsequent values, unless otherwise stated), respectively, on dry basis. The two kinds of commercial NC samples were purchased from Beijing Weixian Nanomaterials Co. Ltd (NC-1) and Jinan Shengquan Group Share Holding Co., Ltd (NC-2), respectively. MB, methyl orange (MO), methyl violet (MV), and congo red (CR) were of analytical grade and supplied by Shanghai McLean Biochemical Co., Ltd. OA dihydrate (≥99.0%) and anhydrous ethanol (≥99.0%) were obtained from Shanghai Aladdin Biochemical Technology Co., Ltd. Polydiallyldimethylammonium chloride (PDADMAC) with molecular weight of 100 kDa was supplied by Sigma-Aldrich Co., Ltd. Hydrochloric acid (HCl, 37%) and sulfuric acid (H_2_SO_4_, 98%) were purchased from Beijing Tongguang Fine Chemical Co., Ltd. Sodium hydroxide (NaOH, >99.5%) was provided by Tianjin Kemio Chemical Reagent Co., Ltd. Deionized (DI) water used in this study was manufactured by using a DW200 deionized water purifier (Shanghai Hetai Instrument Co., Ltd). All chemicals were used as received without further purification.

### 2.2 Preparation of NSR

About 40 g of SR was added into a beaker containing 1 L of DI water. The mixture was mechanically stirred at room temperature at a speed of 200 rpm for 1 h and left to stand for 1 h. The upper liquid was poured out to remove the water-soluble substance. The above steps were repeated for five times. The obtained sediments were dried at 105 °C until a constant weight was reached. Finally, the washed SR was stored in a sealed bag for future use.

The washed SR was pretreated with 10–60% OA aqueous solutions. A typical pretreatment procedure was as follows. Approximately 1.56 g of washed SR was mixed with OA aqueous solution in a three-necked flask, and the solid–liquid mass ratio was 1/80 w/w. Pretreatment was conducted with mechanical stirring (200 rpm) at 110 °C for 3 h. Subsequently, the pretreated SR was washed repetitively with DI water until the washing liquids were detected to be of neutral pH. The pretreated SR was disintegrated to obtain NSR by using an ultrasonic cell crusher (JY96-IIN, Shanghai Huxi Industrial Co., Ltd) under 1 kW and 30 min. The obtained suspension was centrifuged at 3000 rpm for 5 min after ultrasonic disintegration, and the suspension containing NC on the top was collected by removing the precipitate at the bottom. The NSR yield and NC content in NSR were determined using the following equations:1
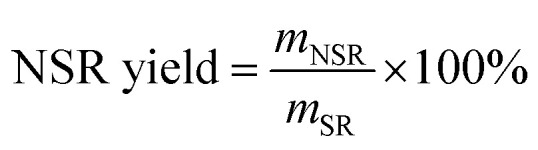
2
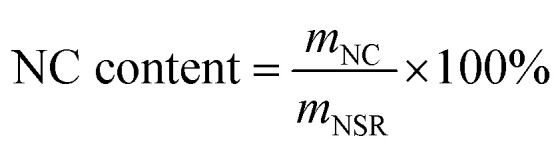
where *m*_NSR_ is the mass of obtained NSR; *m*_SR_ is the mass of used SR; and *m*_NC_ is the mass of prepared NC from SR (SR-NC). The error values were calculated after three repetitions of each reaction.

A series of NSRs was prepared with varying OA contents from 10% to 60%. To simplify the discussion, we devised a notation to indicate the OA content in the pretreatment mixture. For example, the NSR obtained with the OA content of 10% in the pretreatment system was named NSR10, and the resulting NSRs were sequentially named NSR20, NSR30, NSR40, NSR50, and NSR60. For comparison, NSR was also prepared by 5% H_2_SO_4_ pretreatment followed by ultrasonic disintegration, and the obtained NSR sample was labeled as NSR-SA.

### 2.3 Fabrication of MNSR materials

**Scheme 1:**
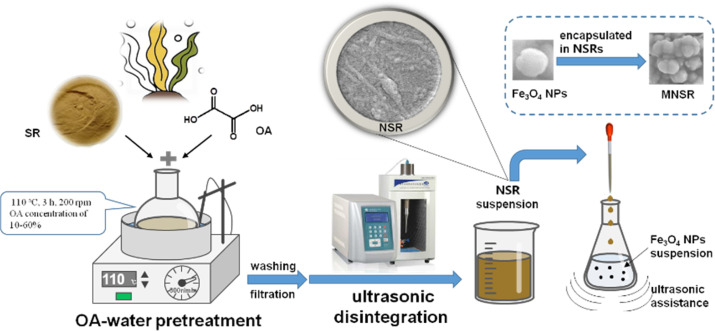
Schematic of MNSR preparation.

The MNSR preparation process is shown in Scheme 1. A series of MNSR samples was prepared by changing the NSR/Fe_3_O_4_ mass ratio of 3/1, 2/1, 1/1, 1/2, or 1/3 w/w, hereinafter referred to as MNSR3/1, MNSR2/1, MNSR1/1, MNSR1/2, and MNSR1/3, respectively. Taking the preparation process of MNSR1/1 as an example, Fe_3_O_4_ NPs (0.3 g) were added into a 250 mL conical flask with 30 mL of DI water and sonicated at 300 W and 40 kHz for 10 min to disperse Fe_3_O_4_ NPs uniformly in the water. About 100 mL of NSR suspension containing 0.3 g of NSR was added dropwise to the Fe_3_O_4_ dispersion in an ultrasonic environment. Thereafter, ultrasonic treatment continued for 10 min. The obtained MNSR1/1 composite materials were separated using an external magnetic force and subsequent high-speed centrifugation (8000 rpm and 10 min). Finally, MNSR1/1 was obtained by freeze-drying at −56 °C for 12 h. The composite process of commercial NC (NC-1 and NC-2) and Fe_3_O_4_ NPs was also investigated to compare the affinity of different kinds of NC and NSR samples.

### 2.4 Characterization of NSR and MNSR

The method established by Wang *et al.*^26^ was used to measure the carboxylic function content per gram of NSR samples for retro titration. All the titrations were carried out in triplicates, and standard deviations were less than ±3.0%. The charge density of the samples was determined at pH 7 with a particle charge detector (PCD 04, BTG Mütek GmbH) using 0.001 M PDADMAC solution as the titrant, as explained elsewhere.^27^

Fourier transform infrared spectrum (FTIR) characterization tests were conducted using the Shimadzu IR Tracer-100 instrument (Japan). The test samples were thoroughly ground, mixed with potassium bromide, and compressed into standard tablets. We selected a spectral range of 500–4000 cm^−1^ for testing and scanned the area 32 times. X-ray polycrystalline diffraction (XRD) analysis was conducted using a Shimadzu XRD-6100 instrument (Japan). Within the range of 10–80°, the Cu target radiation was 40 kV, the current was set to 40 mA, and the test was conducted at a scanning speed of 5° min^−1^. The specific surface area of the NSR samples was measured with the Barrett–Emmett–Teller (BET) N_2_ adsorption method using an Autosorb NOVA2200e volumetric analyzer (Quantachrome, USA).

The morphology and energy-dispersive X-ray spectra (EDS) of the samples were analyzed using an electronic thermal field emission scanning electron microscope (JSM-7800F, Japan), which was equipped with an energy spectrum scanner (X-Mas50, Oxford Instruments). The samples were sprayed with gold before testing. The size distribution of NC in the prepared NSR was determined statistically. A total of 100 samples were randomly selected from the SEM images of each NSR, and their sizes (length and diameter) were measured using Nano Measurer software. Transmission electron microscopy (TEM; Hitachi H-7600) was used to visualize the sample morphology, and it was operated at an accelerating voltage of 100 kV.

During the preparation of magnetic composites, the residual rate of Fe_3_O_4_ NPs was calculated to estimate the affinities of NSR and Fe_3_O_4_ NPs. The residual rate equals the mass percent of unutilized Fe_3_O_4_ NPs. The static magnetic properties of the Fe_3_O_4_ NPs and MNSR samples were characterized and tested using a Lake Shore 7404 vibrating sample magnetometer (VSM, USA). About 30–50 mg of the sample was accurately weighed, and the sample quality was recorded. Before testing, the system was calibrated in three steps: Gaussian meter calibration, magnetic moment cancellation, and magnetic moment gain.^17^ Calibration can eliminate errors caused by sample volume or placement. The surface charge state of Fe_3_O_4_ and MNSR was detected using a zeta potentiometer (Nano ZS, Malvern, the UK). The pH values of the solutions were measured by using a PHS-3C pH meter (Shanghai INESA and Scientific Instrument Co. Ltd).

The adsorption mechanism of MNSR was characterized using an ESCALAB Xi^+^ X-ray photoelectron spectrometer (XPS; Thermo Fisher Scientific, USA). About 15 mg of sample was used for tests under the following conditions: 100 eV full spectrum, 30 eV narrow spectrum, 0.05 eV step size, and residence time of 40–50 ms.

### 2.5 Adsorption characteristics of NSR and MNSR

#### 2.5.1 Adsorption experimental procedure

MB was selected as the target dye to study the adsorption performance of NSR and MNSR. Unless otherwise specified, all adsorption experiments were conducted at the initial pH and 25 °C for 12 h when 30 mg of adsorbent dose was added in 30 mL of dye aqueous solution (100 mg L^−1^). The adsorption experiments were conducted under constant-temperature air oscillation (Changzhou Zhiborui Instrument Manufacturing Co., Ltd) at a frequency of 275 rpm and amplitude of 20 mm. The absorbance changes of MB, MO, MV, and CR dye aqueous solution before and after adsorption at 664, 465, 585, and 497 nm, respectively, were detected by a 50 Scan UV visible spectrophotometer (Varian Technologies China Co., Ltd). The equilibrium adsorption capacity (*q*_e_) and removal efficiency of the adsorbents on dyes were calculated according to eqn (3) and (4):3
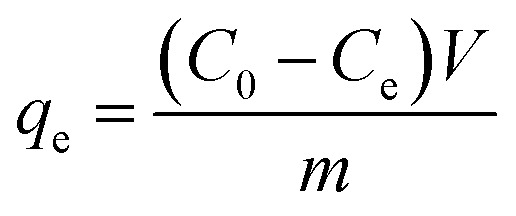
4
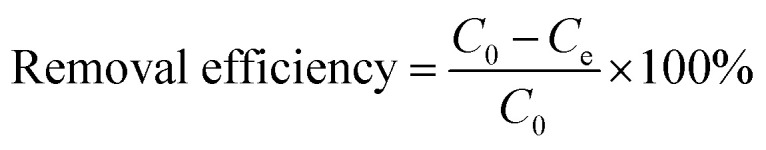
where *C*_0_ (mg L^−1^) is the initial concentration of the dye; *C*_e_ (mg L^−1^) is the adsorption equilibrium concentration; *V* (mL) is the volume of dye aqueous solution; and *m* (g) is the mass of the adsorbent. Each experiment was repeated for at least three times to determine the errors. Statistical analyses were conducted to determine the significance and validity of the experimental data. The mean standard deviation (16.36) and results of paired *t* test within 5% level of significance indicated the success of experiment.^28^

To study the effect of pH, we determined the adsorption of NSR and MNSR in MB aqueous solutions (100 mg L^−1^) with pH from 3 to 10. The pH was adjusted using the aqueous solutions of HCl or NaOH.

#### 2.5.2 Adsorption kinetics

The adsorption behavior of NSR and MNSR was studied within 0–600 min to fit the adsorption kinetics model. This study adopted two adsorption kinetics models, namely, the pseudo first-order model and the pseudo second-order model. The adsorption kinetics models were used to describe the speed of the adsorption process and infer the type of adsorption. The calculation formula and parameter explanations were as follows.

Pseudo first-order model:^20^

5 *q*_*t*_ = *q*_e_(1 − exp^−*k*_1_^)

where *q*_*t*_ (mg g^−1^) represents the adsorption amount at time *t*; *q*_e_ (mg g^−1^) represents the equilibrium adsorption capacity; and *k*_1_ (min^−1^) is a pseudo first-order kinetic rate constant.

Pseudo second-order model:^20^6
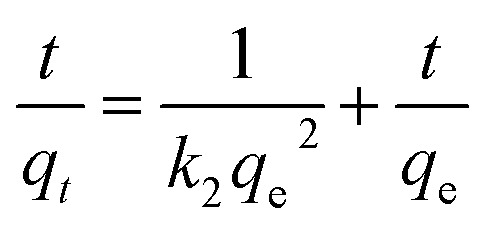
where *q*_*t*_ (mg g^−1^) represents the adsorption amount at time *t*; *q*_e_ (mg g^−1^) represents the equilibrium adsorption capacity; *t* (min) represents the adsorption time; and *k*_2_ (g mg^−1^ min^−1^) is the pseudo second-order kinetic rate constant. Error analysis was conducted by calculating the coefficient of determination *R*^2^ and the value of Akaike Information Criterion (AIC).^29^

#### 2.5.3 Adsorption isotherm

The adsorption effects of NSR and MNSR at a dye concentration of 50–500 mg L^−1^ were studied to fit the adsorption isotherm model. This study used two kinds of adsorption isotherm models, namely, Langmuir and Freundlich. These two isotherms were used to calculate the theoretical maximum adsorption capacity and determine whether the adsorption process belongs to single molecular layer uniform adsorption or uneven multi-layer adsorption. The calculation formula and parameter explanations are as follows.

Langmuir model:^18^7
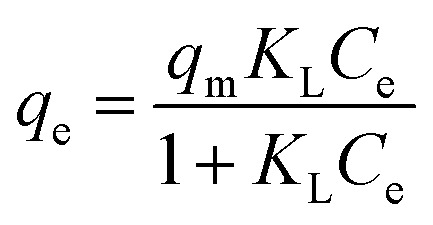
where *q*_e_ (mg g^−1^) represents the equilibrium adsorption capacity; *C*_e_ (mg L^−1^) represents the equilibrium concentration of dye aqueous solution; *q*_m_ (mg g^−1^) represents the theoretical maximum single-layer adsorption capacity of the adsorbent; and *K*_L_ represents the Langmuir equilibrium constant:

Freundlich model:^18^8
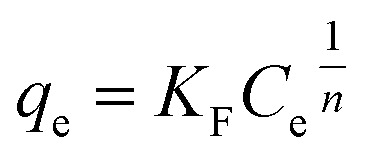
where *q*_e_ (mg g^−1^) represents the equilibrium adsorption capacity; *C*_e_ (mg L^−1^) represents the equilibrium concentration of dye aqueous solution; *K*_F_ represents the equilibrium constant of Freundlich and the multilayer adsorption capacity; and *n* represents the model index. The *R*^2^ and AIC values were calculated to perform error analysis.^29^

#### 2.5.4 Selective adsorption experiments

Four kinds of aqueous solutions of MB, MV, MO, and CR were configured with a dye concentration of 100 mg L^−1^. The two kinds of dye solutions (15 mL) were obtained and mixed in pairs to obtain six sets of mixed dye solutions with a total volume of 30 mL and a total dye concentration of 100 mg L^−1^. About 30 mg of MNSR was added as sorbent to the mixed dye aqueous solutions. The adsorption experiments were conducted according to the procedure described in Section 2.5.1.

#### 2.5.5 Cyclic adsorption experiment

According to the desorption methods reported in the literature,^26,27^ 0.05 M HCl aqueous solution (hereinafter referred to as HCl) and anhydrous ethanol were used as the desorption agents for the MNSRs. Specifically, after completing the adsorption experiment in 100 mg per L MB aqueous solution, the adsorption materials were separated from the MB aqueous solution by using an external magnetic field. The separated MNSRs were added in 30 mL of desorption agent, and the desorption experiment was conducted at 25 °C for 12 h in a constant-temperature air oscillator. Solid–liquid separation was repeated to recover the MNSRs after desorption. The recovered MNSRs were freeze-dried and used in the next cycle of the adsorption experiment. The regeneration efficiency (RE) was calculated according to the following formula:9
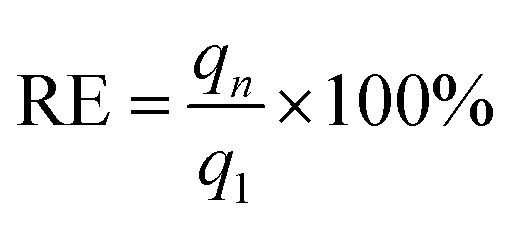
where *q*_1_ (mg g^−1^) represents the equilibrium adsorption amount for the first adsorption; and *q*_*n*_ (mg g^−1^) represents the equilibrium adsorption amount for the *n*th time.

## 3. Results and discussion

### 3.1 Yield of NSR

**Fig. 1:**
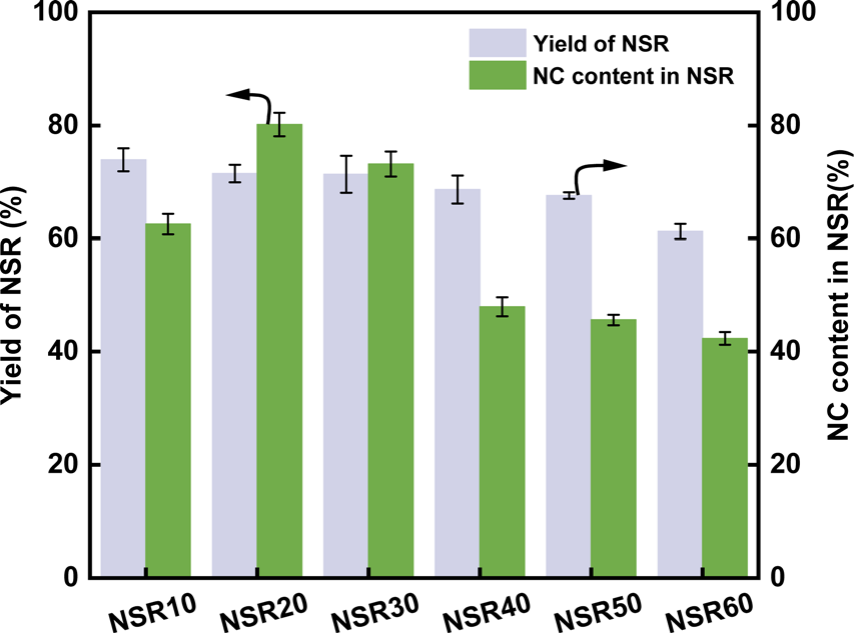
Yield of NSR and NC content in NSR.

The yield of NSR prepared from SR is indicated in Fig. 1. The yield of NSR monotonously decreased from 73.9% to 61.2% with increasing OA content in pretreatment systems from 10% to 60%. The hydrolysis of cellulose in SR promoted by OA–water systems cleaved part of β-1,4-glycosidic linkages, which resulted in the breakup of cellulose chains.^22^ The addition of more OA in the pretreatment systems resulted in the release of more H^+^ ions, which improved the hydrolysis of cellulose. Although hydrolysis was favorable for the dissociation of cellulose fibers in the SR, a part of cellulose could be converted into reducing sugars, which resulted in the decrease in the NSR yields. In addition, SR is rich in ash (about 24.7%). The high OA content in the pretreatment systems could be favorable for the removal of ash from SR, thereby reducing the NSR yield.

The NC content in NSR illustrates the nanorization degree of NSR, as shown in Fig. 1. With increasing OA content in pretreatment systems from 10% to 60%, the NC content first increased and then continuously decreased. In particular, at the high OA concentration (40–60%), the NC content in NSR decreased to 47.9–42.3%. The high OA concentration could lead to the excessive hydrolysis of SR cellulose, which contributed to the decrease in NSR–NC production. Moreover, the high OA concentration could favor the cross-linking of cellulose fragments through esterification^30^. The highest NC content of 80.2% was obtained when the SR was pretreated with the 20% OA–water system. This result suggested that NSR20 had a high nanorization degree.

### 3.2 Characterization of NSR

#### 3.2.1 Surface morphology

The surface morphology of SR is shown in Fig. S1.† SR was in the form of a block (around 10–100 μm). The surface morphologies of the prepared NSR samples are shown in Fig. S2.† The blocks of SR (Fig. S1†) were individually broken up into micro- and nano-sized cellulose fibers/particles. Notably, some SR blocks were still not broken up even after OA–water pretreatment followed by ultrasonic disintegration, particularly NSR10. We observed that the amount of residual SR blocks in NSR20 (Fig. S2c†) and NSR60 (Fig. S2g†) was lower than that in the other prepared NSR samples (Fig. S2a, S2b, S2d, and S2e†), indicating that majority of the SR blocks were dissociated into nanosized fibers/particles. As shown in Fig. S3,† the EDS spectra indicated that the obtained SR NPs were complexes containing cellulose and ash. Furthermore, the prepared NSRs contained a large number of NSR–NC, which are primarily responsible for the encapsulation of Fe_3_O_4_ NPs.

#### 3.2.2 Size distribution of NSR–NC

The size distributions of the prepared NSR–NC samples are shown in Fig. S4.† With increasing OA concentration in the pretreatment system from 10% to 60%, the length and diameter distributions of the prepared NSR–NC gradually became narrow (Fig. S4a and b†). The hydrolysis of cellulose resulted in the destruction of SR cellulose chains with the help of the OA–water systems.^31^ Pretreatment with an OA–water system of high OA content favored the dissociation of SR cellulose bundles and shortening of cellulose fibers. With increasing OA content from 10% to 60%, the average lengths and diameters nearly monotonously decreased from 910 and 48 nm to 595 and 32 nm. NC in NSR60 had the minimum average length and diameter among the prepared NSR–NC samples (Fig. S4c†). These findings indicated that the high nanorization degree of SR was achieved in the case of OA–water pretreatment with OA concentration from 10% to 60%, which was in accordance with the results of SEM characterization (Fig. S2†).

#### 3.2.3 FTIR analysis

The FTIR spectra of the prepared NSR samples are presented in Fig. S5.† The bands at 1111 and 1034 cm^−1^ represented the stretching of C–O of cellulose and C–O–C on the pyranose ring, respectively. The band at 1384 cm^−1^ was the characteristic vibrational stretching of C–O bonds in the polysaccharide rings. These typical bands attributed to the cellulose structure were found in the FTIR spectra, suggesting that SR and prepared NSRs were composed of cellulose. Compared with the spectrum of SR, the stretching bands of the hydroxyl (O–H) groups of NSR samples shifted from 3441 cm^−1^ to 3423 cm^−1^, indicating a change in the type of O–H bonds in cellulose molecules.^32^ The intensities of ester carbonyl (C=O) stretching (1734 cm^−1^) and carboxyl (C=O) stretching (1624 cm^−1^) of the NRS samples showed a slight increase, which could be due to the esterification reaction between cellulose and OA. In particular, this reaction was one of the reasons for the change in the type of –OH groups.

#### 3.2.4 Carboxyl group content

Under heating conditions (*i.e.*, 110 °C), OA will dehydrate to yield a reactive anhydride, which can react with hydroxyl groups on SR to form carboxylated NSR (Scheme S1†). The carboxyl group content of unpretreated SR was 1.79 mmol g^−1^. With increasing OA concentration in pretreatment systems from 10% to 60%, the carboxyl group content of NSR samples monotonously increased (Table S1†) and followed the order: NSR60 (6.73 ± 0.38 mmol g^−1^) > NSR50 (6.26 ± 0.29 mmol g^−1^) > NSR40 (6.01 ± 0.26 mmol g^−1^) > NSR30 (4.96 ± 0.19 mmol g^−1^) > NSR20 (4.74 ± 0.21 mmol g^−1^) > NSR10 (4.58 ± 0.13 mmol g^−1^). Pretreatment with an OA–water system of a high OA content favored the increase in carboxyl group content of NSR samples. This result was consistent with the findings of FTIR analysis. The high content of OA enhanced the probability for contact between SR cellulose and reactive anhydride derived from OA.

#### 3.2.5 XRD analysis

The crystalline structures of the prepared NSR samples were explored by XRD within a scanning range of 10° < 2*θ* < 70°, and the results are shown in Fig. S6.† No peaks were found in the diffraction patterns around 2*θ* = 19.7°, which was commonly assigned to the less ordered or amorphous region of the cellulose chains. Three characteristic diffraction peaks were located around 14.8°, 22.8°, and 24.3° in the XRD patterns of NSRs, which were attributed to the crystal planes of cellulose.^33^ The major crystalline peak was recorded at 24.3°, confirming the presence of typical cellulose-I crystalline structures.^34^

### 3.3 Adsorption characteristics of NSR

**Fig. 2:**
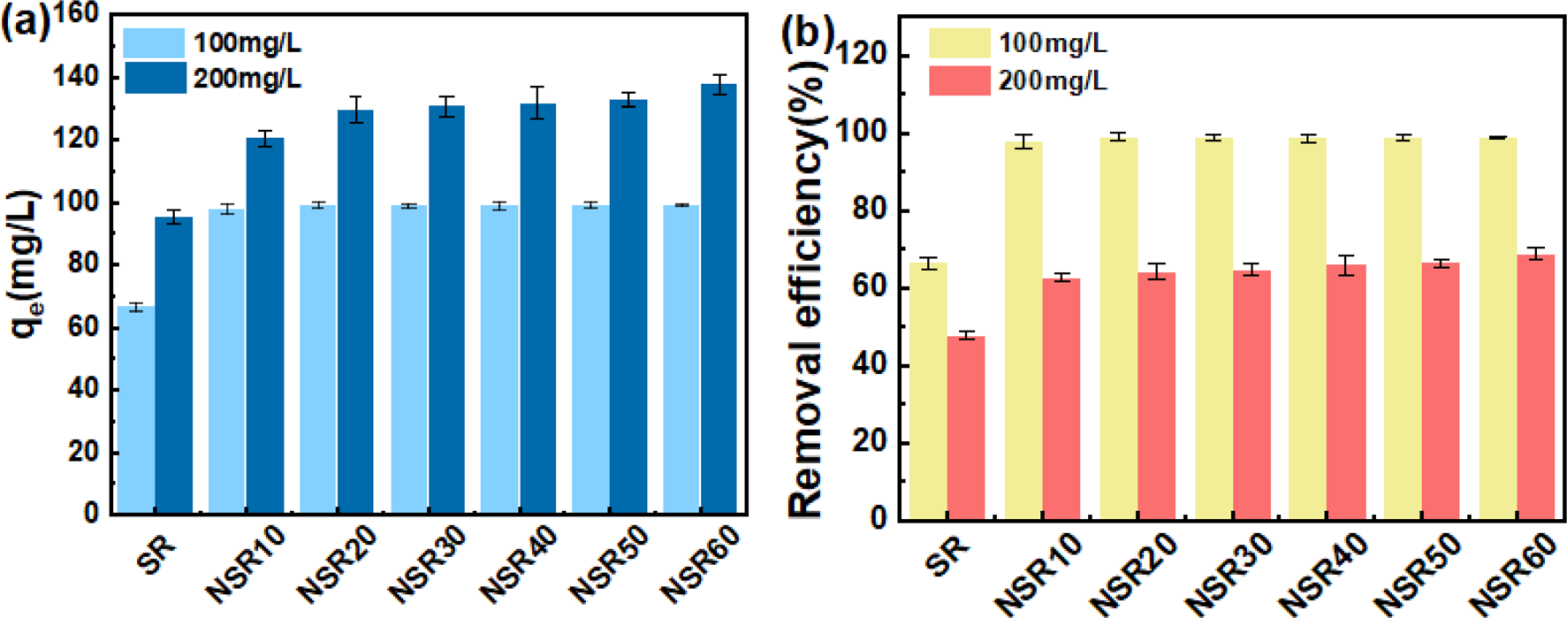
*q*_e_ (a) and removal efficiency (b) of SR and prepared NSRs for MB adsorption.

The equilibrium dye uptake of SR and the prepared NSRs under MB dye contents of 100 and 200 mg L^−1^ is indicated in Fig. 2. *q*_e_ of the NSR samples was clearly higher than that of SR. For example, at an MB content of 200 mg L^−1^, *q*_e_ of NSRs was 125.42–137.57 mg g^−1^, whereas that of SR was only 95.35 mg g^−1^ (Fig. 2a). The –OH groups on cellulose materials were the main functional active sites for MB adsorption through the formation of hydrogen bonds and electrostatic interaction.^35^ More –OH groups on NSR could be exposed after nanorization compared with that on SR. In addition, as shown in Table S1,† the measured *S*_BET_ values of NSR samples were 57.60 (NSR10), 48.89 (NSR20), 43.54 (NSR30), 62.03 (NSR40), 58.84 (NSR50), and 57.38 m^2^ g^−1^ (NSR60), which were commonly higher than that of SR (36.91 m^2^ g^−1^). The disassociation of cellulose fiber bundles in SR resulted in a high specific surface area of NSR, which favored MB dye adsorption.

At an MB content of 100 mg L^−1^, we found no clear difference among the prepared NSR samples for *q*_e_ (around 98 mg g^−1^), but the removal efficiency exceeded 98% (Fig. 2b). This result indicated that MB in the aqueous solutions had nearly been completely adsorbed by the NSRs. When the MB content increased to 200 mg L^−1^, the removal efficiency of 62.71–68.79% was obtained for all the NSRs (Fig. 2b). The prepared NSRs were favorable for MB removal at a low dye content (*i.e.*, 100 mg L^−1^).

Notably, no obvious difference in *q*_e_ was observed at an MB content of 200 mg L^−1^. For example, the *q*_e_ values of NSR10 and NSR20 were 132.76 and 137.57 mg g^−1^, respectively, whereas those of NSR50 and NSR60 were 128.36 and 131.77 mg g^−1^, respectively. The adsorption capacity of NSR samples was not correlated with the size distribution of NSR-NC (Fig. S4†) and carboxyl group content. It could be attributed to the complexity of influencing factors of MB adsorption on the NSRs, including active sites, NC content, and microstructure.

The NSR material used in preparing MNSR was selected after evaluating the cost of OA utilization and its adsorption capacity. The adsorption capacity of NSR20 was not lower than that of other NSR samples (Fig. 2). Moreover, the yield of NSR20 was higher than that of other NSR samples (Fig. 1). A low OA content (20%) in the pretreatment system was used in the preparation process, which provided an opportunity to obtain NSR20 materials at low cost. A high NC content in NSR20 was also favorable for the encapulsation of Fe_3_O_4_ NPs. Therefore, NSR20 was chosen as the material to prepare MNSRs in subsequent experiments.

### 3.4 Characterization of MNSR

#### 3.4.1 Affinities between NSR20 and Fe_3_O_4_ NPs

**Fig. 3:**
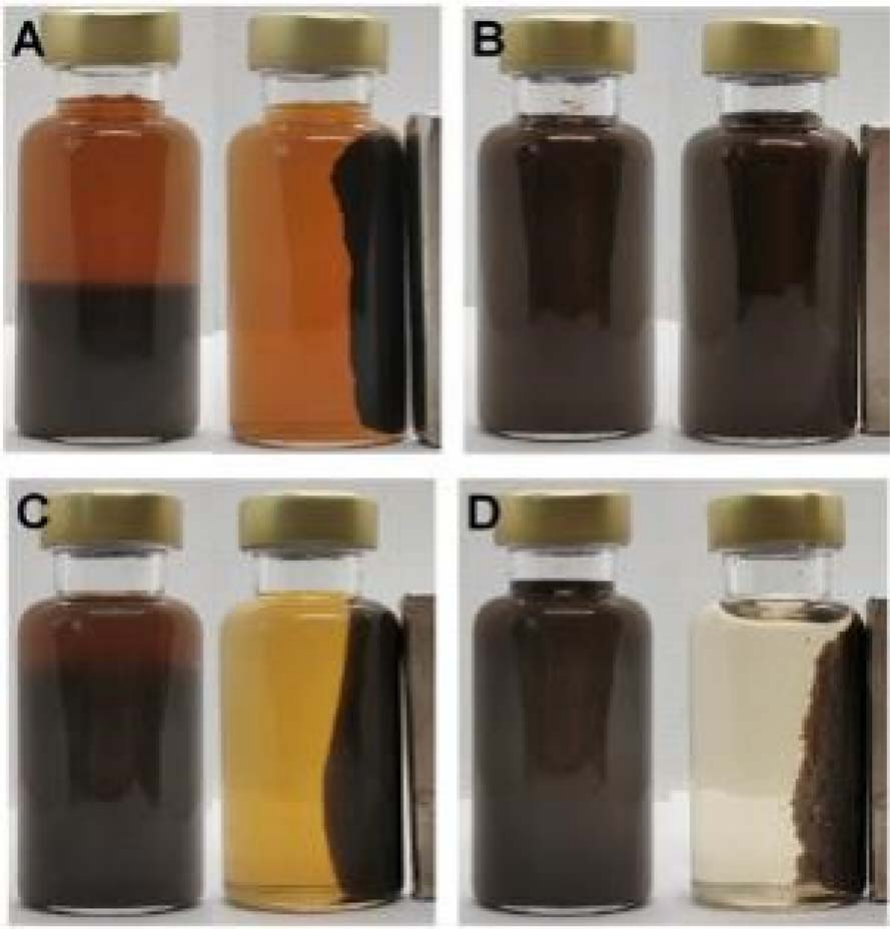
Images of magnetic composite materials prepared from Fe_3_O_4_ NPs and NC-1 (A), NC-2(B), NSR-SA5 (C), and NSR20 (D).

The affinities between NSR20 and Fe_3_O_4_ NPs were investigated by comparison with other NC materials. When the obtained dispersions of NC/Fe_3_O_4_ NPs were placed in an external magnetic field, the color differences of the liquid phase could be clearly observed (Fig. 3). In this case, the colors of the solutions were brown (NC-1/Fe_3_O_4_), nontransparent brown black (NC-2/Fe_3_O_4_), light brown (NSR-SA/Fe_3_O_4_), and faint yellow (NSR20/Fe_3_O_4_). In the case of preparing magnetic composites with different NC materials, the residual rate of Fe_3_O_4_ NPs followed the order (Table S2†): NC-2 (46.3%) > NC-1 (18.9%) > NSR-SA (8.5%) > NSR20 (1.4%). Thus, the composite material did not form through NC-2/Fe_3_O_4_ interactions. NC-2 was prepared by the mechanical method, and the long fibers with length of more than 1 μm (Fig. S7b†) could result in the encapsulation difficulty of Fe_3_O_4_ NPs. By contrast, the lengths of NC-1 (Fig. S7a†), NC of NSR-SA (Fig. S7c†), and NC of NSR20 (Fig. S7d†) were smaller than 1 μm, which helped their interactions with Fe_3_O_4_ NPs.

As shown in Fig. 3, NSR20 had the best affinity with Fe_3_O_4_ NPs compared with the other materials. For the above used materials, the density of surface charge was in the following order (Table S2†): NC-2 (−13 ± 2 μeq. g^−1^) < NC-1 (−42 ± 5 μeq. g^−1^) < NSR-SA (−87 ± 3 μeq. g^−1^) < NSR-20 (−350 ± 12 μeq. g^−1^). The carboxylation of NSR20 through the esterification of OA enhanced negative electricity, which was favorable for the electrostatic interactions between NSR20 and Fe_3_O_4_ NPs with positive charge. In addition, the affinities of NSR20 and NSR-SA prepared from the SR were better than those of NC-1 and NC-2 derived from other cellulose sources, which could be due to the formation of charged functional groups in SR during seaweed processing. Thus, SR has application potential in the preparation of composite materials.

#### 3.4.2 SEM and TEM analysis

**Fig. 4:**
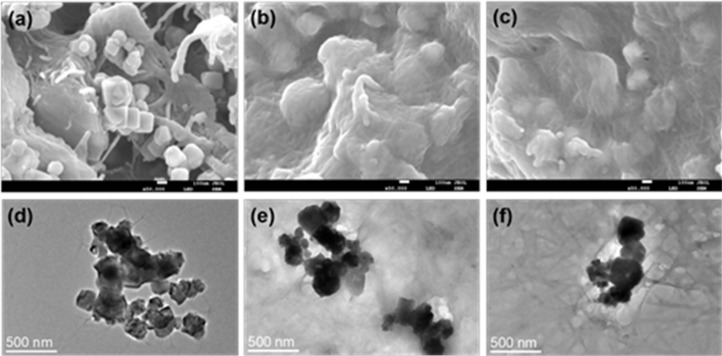
SEM images of prepared MNSR1/2 (a), MNSR1/1 (b), and MNSR2/1 (c) and TEM images of MNSR1/2 (d), MNSR1/1 (e), and MNSR2/1 (f).

The microstructures of the prepared MNSRs are shown in Fig. 4. The average diameter of cuboidal Fe_3_O_4_ NPs was about 50–350 nm. For MNSR1/2 (Fe_3_O_4_ content of 66.7%), some Fe_3_O_4_ NPs were visible with SEM and TEM (Fig. 4a and d), and they were not covered by the NSR network. When the Fe_3_O_4_ content in the composite decreased to 50% (thereby increasing the NSR content to 50.0%), the Fe_3_O_4_ NPs were fully encapsulated in the NSR aggregates (MNSR1/1, Fig. 4b). The encasing cellulose layer of MSNR2/1 with Fe_3_O_4_ content of 33.3% was thicker than that of MNSR1/1 (Fig. 4c). Moreover, the TEM images (Fig. 4e and f) demonstrated that the NSR aggregates (grey) completely covered the Fe_3_O_4_ NPs (dark).

#### 3.4.3 Zeta potential

To prepare MNSR composites, we added positively charged Fe_3_O_4_ NP aqueous solution (+19.7 mV) dropwise to the NSR dispersion (−25.2 mV) *via* ultrasonic assistance. At the NSR20 contents of 33.3% (MNSR1/2), 50.0% (MNSR1/1), and 66.7% (MNSR2/1), the zeta potentials of the mixing systems were −21.1, −22.5, and −22.6 mV, respectively. The changes in zeta potentials indicated the combination of NSR20 and Fe_3_O_4_ NPs *via* electrostatic interaction. For the MNSR composites with a high NSR20 content (50.0–66.7%), the zeta potentials hardly changed, suggesting that the Fe_3_O_4_ NPs were fully covered by the NSR20 aggregates. This finding was consistent with the morphology presented in SEM and TEM images (Fig. 4).

#### 3.4.4 Magnetic properties

**Fig. 5:**
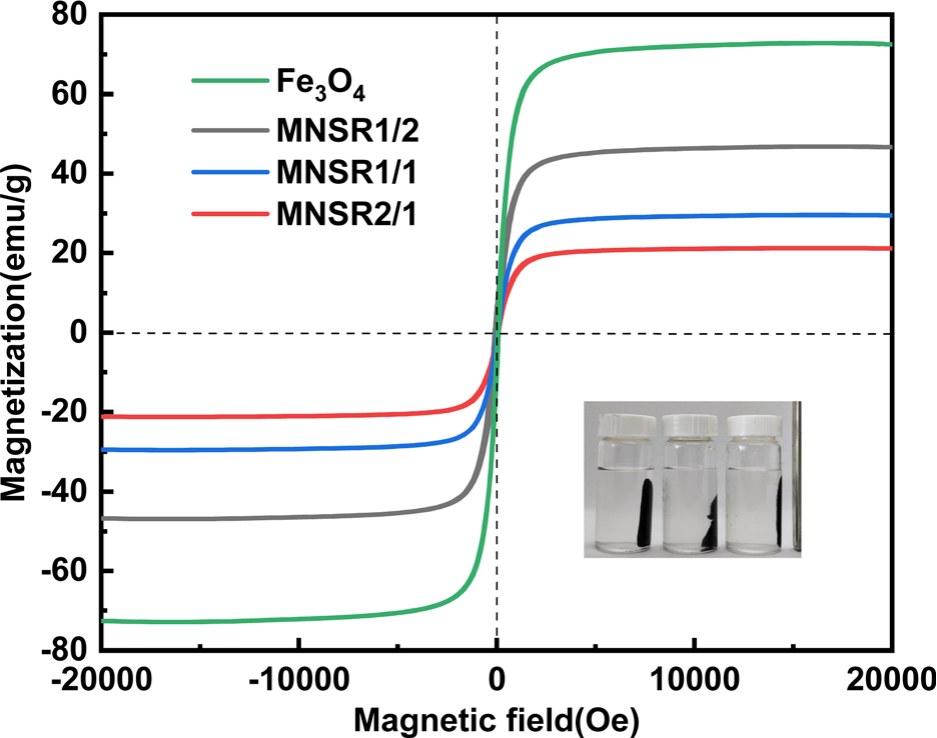
Magnetization curves of Fe_3_O_4_ NPs and prepared MNSR samples.

The magnetic properties of Fe_3_O_4_ NPs and prepared MNSR samples are shown in Fig. 5. The saturation magnetization of Fe_3_O_4_ NPs was 72.5 emu g^−1^, whereas that of the prepared MNSRs was 21.1–46.7 emu g^−1^. The saturation magnetization of prepared MNSRs was positively correlated with the content of Fe_3_O_4_ NPs in the composites. All the magnetization curves went through the zero point where the remanence and coercivity were equal to zero, indicating the superparamagnetic characteristics of these prepared MNSR composites.^36^ The MNSRs, as the adsorbents, could be easily separated from wastewater with an external magnetic field, which improved the recyclability of MNSRs.

### 3.5 Adsorption behavior of MNSRs

#### 3.5.1 Influencing factors for MB adsorption

**Fig. 6:**
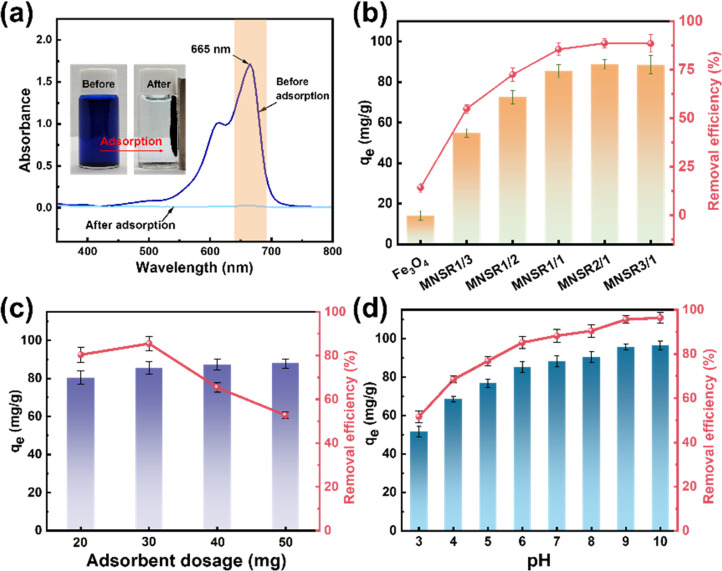
Images of UV-vis spectra before and after adsorption (a), effects of adsorbent type (b), dosage of MNSR1/1 (c), and pH (d) on *q*_e_ and the removal efficiency for MB adsorption.

The effects of adsorbent type, adsorbent dosage, and pH on the equilibrium of dye uptake are illustrated in Fig. 6. At an initial MB content of 100 mg L^−1^ and the adsorbent dosage of 30 mg, *q*_e_ of Fe_3_O_4_ NPs (14.21 mg g^−1^) was far lower than that of the prepared MNSR (Fig. 6a). Moreover, *q*_e_ of the prepared MNSRs was lower than that of NSR20 (98.92 mg g; Fig. 2a). As the main component containing active sites, the amount of NSR in MNSR was less than that in pure NSR20. Meanwhile, the capture of Fe_3_O_4_ NPs could lead to the partial blockage of adsorption sites on NSR20.^21^

When the mass ratio of NSR/Fe_3_O_4_ was raised from 1/3 to 1/1, *q*_e_ increased from 54.89 mg g^−1^ (MNSR13) to 85.48 mg g^−1^ (MNSR1/1), as shown in Fig. 6a. However, when the mass ratio of NSR/Fe_3_O_4_ continued to increase to 2/1 or 3/1, *q*_e_ and the removal efficiency of MNSR (MNSR2/1 or MNSR3/1) almost did not increase (∼88 mg g; Fig. 6a). This finding may be due to the complete encapsulation of Fe_3_O_4_ NPs by the NSR20 aggregates. The superposition of NSR fibrils limited the number of active sites where adsorption occurred. Moreover, the magnetic property of MNSR1/1 was stronger than that of MNSR2/1 (Fig. 5), which favored the recovery and recycling of MNSR. Thus, MNSR1/1 was selected in the following experiments.

As shown in Fig. 6b, as the dosage of MNSR1/1 increased from 20 mg to 30 mg, *q*_e_ increased from 80.39 mg g^−1^ to 85.48 mg g^−1^, but then it sharply decreased to 65.44 mg g^−1^ and 52.73 mg g^−1^ at the adsorbent dosage of 40 or 50 mg. The MB removal efficiency of 85.5–87.8% was achieved at the MNSR1/1 dosage of 30–50 mg, suggesting that MB adsorption reached the saturation state. Unfortunately, under the experimental conditions, the complete removal of MB from the aqueous solutions seemed to be impossible because of the limitations of the adsorption–desorption equilibrium of MNSR1/1.

The pH of aqueous solutions influences the surface charge of the adsorbent and causes variations in the adsorption capacity for the dye.^37^Fig. 6c shows the variation tendency of *q*_e_ and removal efficiency of MB dye (100 mg L^−1^, initial pH 6.6) with varying pH from 3 to 10. As the pH of the system increased (pH 3–10), *q*_e_ and the removal efficiency of MB by MNSR1/1 monotonously increased. At pH 10, the maximum removal efficiency of MNSR1/1 (96.3%) was achieved. Under acidic conditions (pH 3–5), the removal efficiency (51.6–76.8%) was relatively low due to protonation and electrostatic repulsion. The active sites on the adsorbent are susceptible to protonation, resulting in their electropositive nature. In this work, pH 6 was deemed close to the initial pH of MB aqueous solution. The decrease in the H^+^ concentration led to a decrease in the protonation degree of the active sites on the adsorbent, which weakened the electrostatic repulsion effects. This phenomenon was advantageous for capturing MB *via* mutual electrostatic attraction, thereby improving the adsorption capacity. The significant increase in the removal efficiency of MB dye by MNSR1/1 at pH 7–8 was due to the enhanced electronegativity of surface charges under alkaline conditions, which facilitated the adsorption of cationic dyes.^37^ At pH 9 or 10, the removal efficiency of MB peaked (95.6% or 96.3%, respectively), possibly due to the saturation of negatively charged active sites on the surface of cellulose. These results indicated that the alkaline conditions were strongly favorable for the adsorption of MB by MNSR1/1.

#### 3.5.2 Adsorption kinetics

**Fig. 7:**
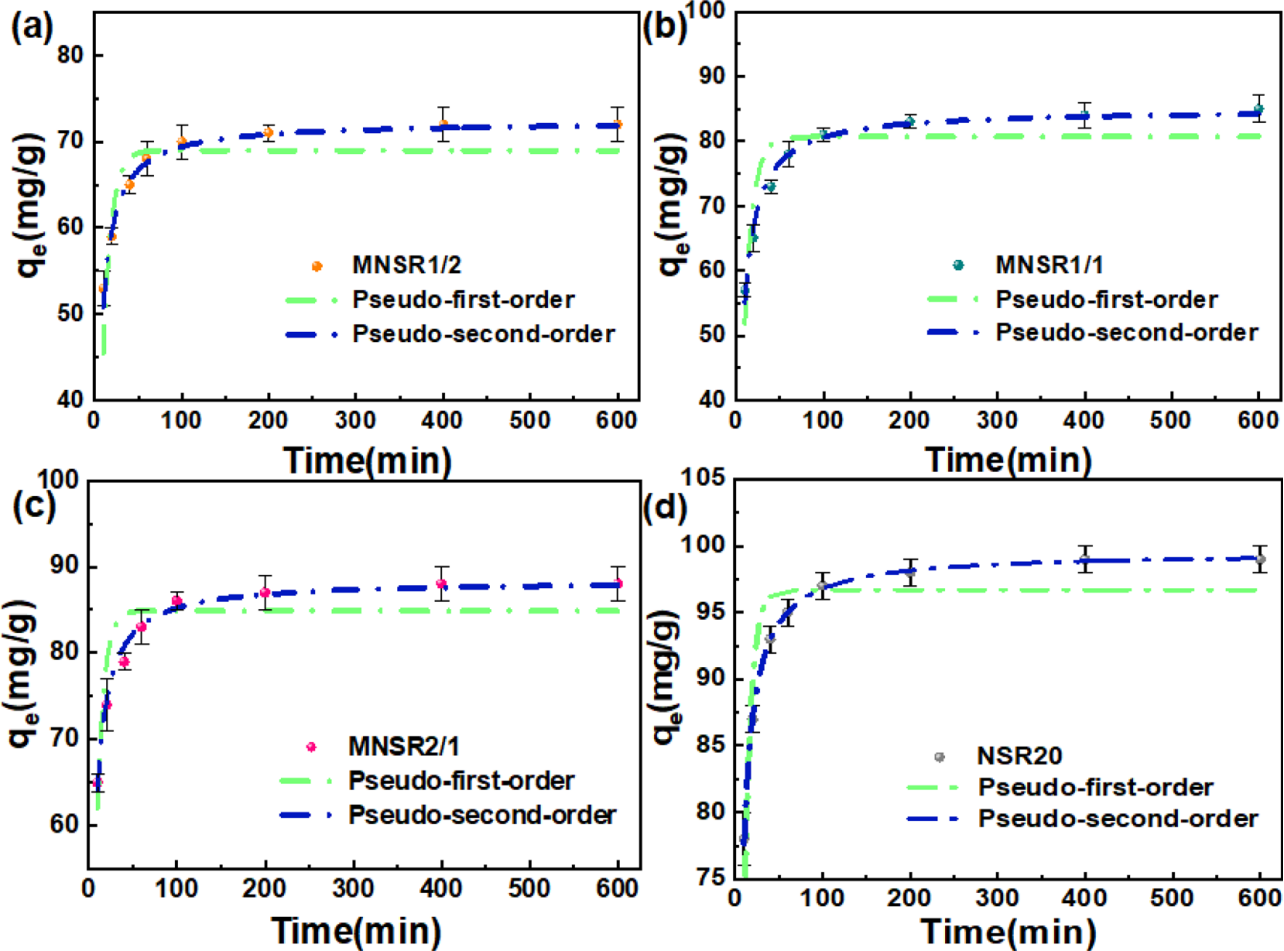
Adsorption kinetics of MB on MNSR1/2 (a), MNSR1/1 (b), MNSR2/1 (c), and NSR20 (d).

To investigate the influence of contact time on the adsorption process of MB dye on the MNSR1/2, MNSR1/1, MNSR2/1, and NSR20 adsorbents, we used the pseudo-first-order kinetic model and pseudo-second-order kinetic model for fitting. The fitting results are shown in Fig. 7 and Table 1. The MB adsorption capacities of the four kinds of adsorbents increased sharply within 100 min, followed by a gradual approach toward the maximum adsorption capacity due to the improved adsorption process originating from the high ratio of adsorbing sites to adsorbates; this phenomenon was followed by a slow increase until adsorption equilibrium was reached.^15^ After 100 min, the reduction of effective active sites limited the adsorption rate. According to the results of kinetic fitting, the adsorption of MB on MNSRs and NSR20 followed the pseudo-second-order kinetic model, with a high correlation coefficient *R*^2^ of 0.985–0.999 and low AIC values of 13.95–29.13. Thus, chemisorption is believed to play a dominant role in the adsorption process.^38,39^

**Table 1:** Kinetic parameters of MB adsorption on the MNSRs and NSR20

Adsorbents	Pseudo-first-order model				Pseudo-second-order model			
	*q*_e_ (mg g^−1^)	*K*_1_ (min^−1^)	*R* ^2^	AIC	*q*_e_ (mg g^−1^)	*K*_2_ (g mg^−1^ min^−1^)	*R* ^2^	AIC
MNSR1/2	68.97	0.108	0.739	74.19	72.35	0.003	0.985	24.45
MNSR1/1	80.69	0.103	0.807	50.43	84.96	0.002	0.984	29.13
MNSR2/1	84.89	0.131	0.811	35.70	88.42	0.003	0.990	15.51
NSR20	96.70	0.132	0.752	60.39	99.54	0.004	0.999	13.95

#### 3.5.3 Adsorption isotherms

**Fig. 8:**
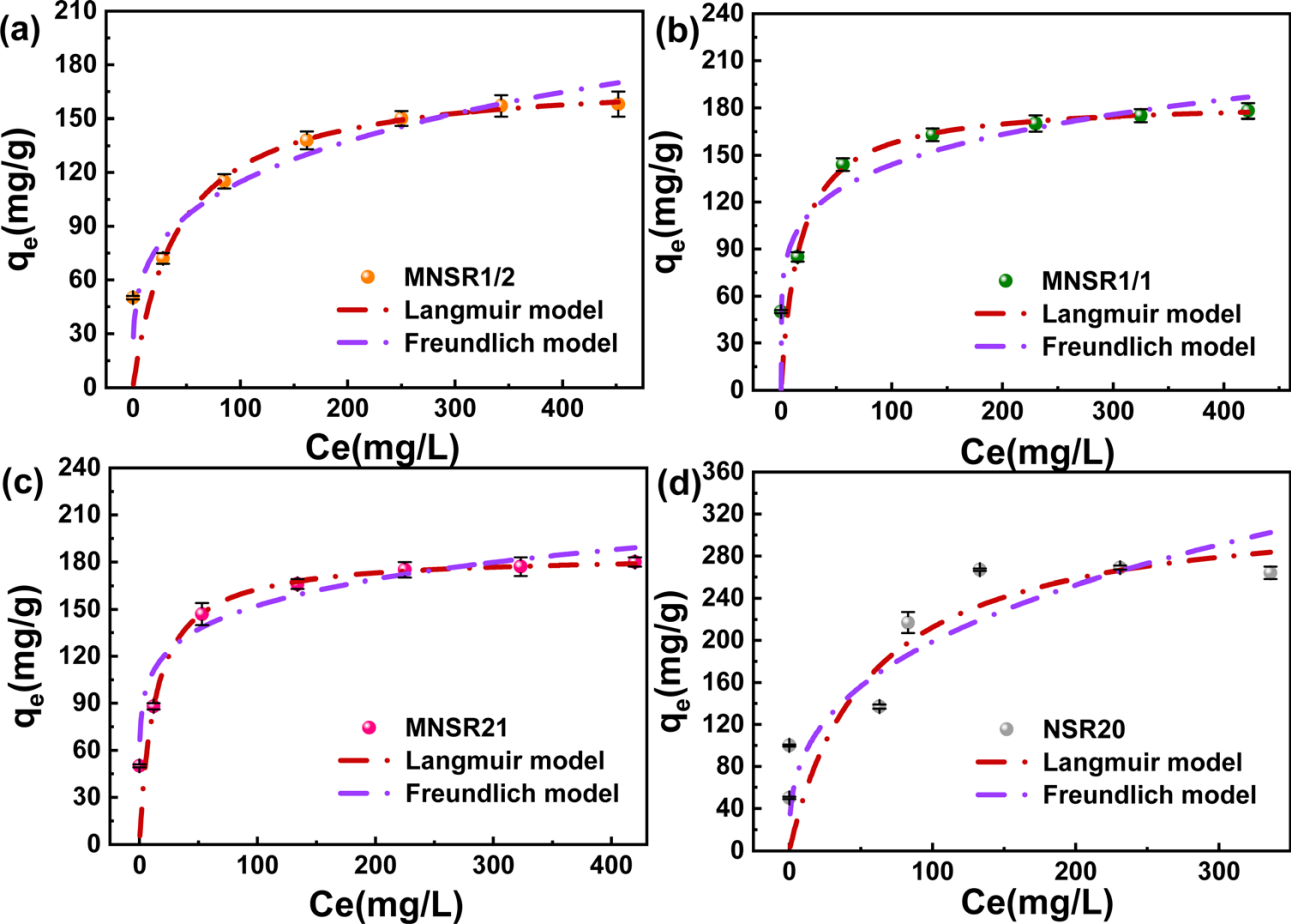
Adsorption isotherms of MB on MNSR1/2 (a), MNSR1/1 (b), MNSR2/1 (c), and NSR20 (d).

The Freundlich and Langmuir isotherm models are commonly used to explain the physicochemical adsorption phenomena of cationic dyes in their interaction with the surface of adsorption materials.^40^ The fitting results of the two isothermal models for the adsorption of MB dye on MNSR1/2, MNSR1/1, MNSR2/1, and NSR2/0 are shown in Fig. 8 and Table 2. The adsorption effect of MNSRs did not significantly improve with the increase in dye concentration from 200 mg L^−1^ to 400 mg L^−1^. For the three adsorbents, the correlation coefficients for the Langmuir models (*R*^2^, 0.998–0.999) were obviously higher than those for the Freundlich models (*R*^2^, 0.790–0.921), whereas AIC values for the isotherm models showed the opposite trend. It suggested that the Langmuir isotherms were sufficiently matched to describe the adsorption characteristics. Therefore, the adsorption of MB on MNSRs could be described as a surface monolayer with a limited number of adsorption sites.^41^ The high *R*^2^ and low AIC value for Freundlich models illustrated that the adsorption process of MB by NSR20 was not a single-layer adsorption on the surface but a multi-layer adsorption process with an uneven surface.^42^

**Table 2:** Absorption isotherms of MB on MNSR1/2, MNSR1/1, MNSR2/1, and NSR20

Materials	Langmuir isotherm				Freundlich isotherm			
	*q*_m_ (mg g^−1^)	*K*_L_ (mg L^−1^)	*R* ^2^	AIC	*K*_F_ (mg g^−1^)	*n*	*R* ^2^	AIC
MNSR1/2	173.67	0.024	0.998	12.63	34.59	3.840	0.921	45.69
MNSR1/1	184.25	0.059	0.998	12.64	62.29	5.533	0.790	66.68
MNSR2/1	184.50	0.075	0.999	7.88	76.10	6.636	0.830	64.73
NSR20	330.49	0.018	0.648	72.60	40.36	2.890	0.953	36.37

The maximum adsorption capacity (*q*_max_) of MNSR1/1 for MB reached 184.25 mg g^−1^*q*_max_ of MNSR1/1 for MB adsorption was compared with other adsorbents that have been reported in the literature, which further evaluated the adsorption performance of MNSR1/1 (Table S3†). The research suggested that MNSR1/1 can effectively remove MB dye from the aqueous solution at a initial pH at 25 °C. Thus, MNSR1/1 is a promising adsorbent for wastewater purification.

#### 3.5.4 Competitive adsorption

**Fig. 9:**
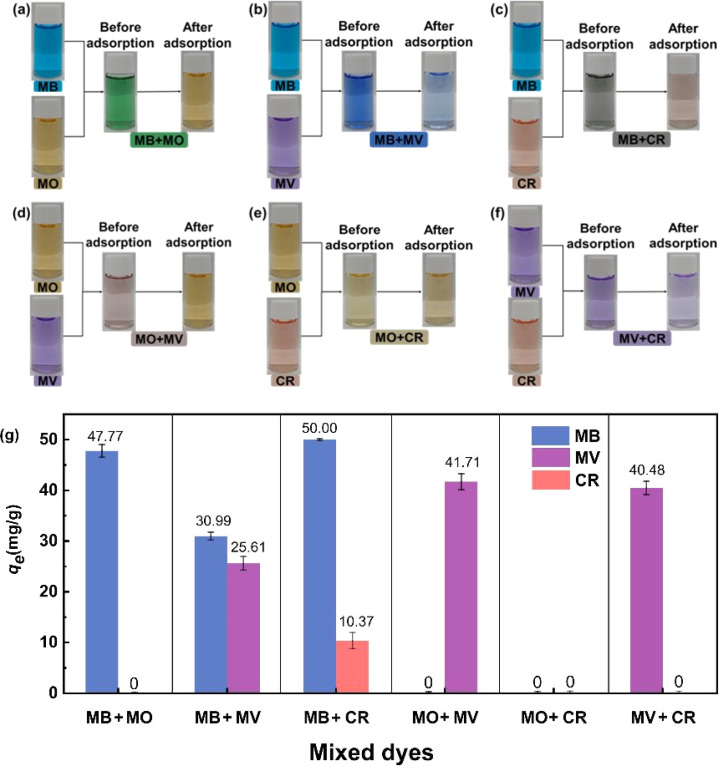
Photographs showing the selective adsorption of MB + MO (a), MB + MV (b), MB + CR (c), MO + MV (d), MO + CR (e), and MV + CR (f) dye mixture on MNSR1/1; the obtained *q*_e_ in competitive adsorption (g).

Other organic dyes also exist in wastewater, so the competitive adsorption between coexisting organic dyes must be explored. The adsorption characteristics of MNSR1/1 in the MB + MO, MB + MV, MB + CR, MO + MV, MO + CR, and MV + CR systems were investigated, as shown in Fig. 9 and S8.† The color of the only anionic dyes (MO or CR) was observed after adsorption by MNSR1/1 in the case of dye mixtures of MB + MO (Fig. 9a), MB + CR (Fig. 9c), MO + MV (Fig. 9d), or MV + CR (Fig. 9f). Thus, the cationic dyes (MB or MV) could be efficiently and selectively removed from the aqueous solutions of cationic dyes coexisting with anionic dyes (MO or CR).

As shown in Fig. 9g, *q*_e_ for adsorbing cationic dyes (MB and MV) was significantly superior to that for adsorbing anionic dyes (MO and CR) in these coexisting dye mixtures. This finding confirmed that MNSR1/1 exhibited the selective adsorption capacity for cationic dyes (*i.e.*, MB and MV) from the aqueous solutions. This phenomenon could be caused by the negative charge on the surface of MNSR1/1, which was consistent with the results of zeta potential analysis. MNSRs exert electrostatic attraction on positively charged cationic dyes and electrostatic repulsion on negatively charged anionic dyes.^43^

#### 3.5.5 Recyclability of MNSR

Reusability is an important factor in evaluating the performance of adsorbents. Therefore, the cycling ability of MNSR1/1 to adsorb MB was investigated. Fig. S9† shows that the RE of MNSR1/1 for MB adsorption monotonously decreased with increasing cycling times. This result was due to the loss of active sites in cellulose during desorption, or it may be related to a slight decrease in material quality and incomplete desorption during the cycling experiments.

As shown in Fig. S9,† the type of desorption agents (0.05 M HCl or ethanol) influenced the RE values of MNSR1/1, which may be due to the differences in their desorption role. After the fifth cycle of adsorption–desorption experiment, the RE with 0.05 M HCl as desorption agent was 82.71%, whereas that with ethanol as desorption agent was 77.62%. Thus, the desorption capacity of 0.05 M HCl was better than that of ethanol for MNSR1/1. The recycling experimental results suggested that the stability of MNSR1/1 could be utilized repeatedly under practical situations for the treatment of wastewater.

### 3.6 Possible adsorption mechanism

The MNSR1/1 samples before and after adsorption were characterized by EDS, FTIR, and XPS to explore the possible adsorption mechanism. Fig. S10† displays the detection results for the distribution of elements in MNSR1/1. According to the spectra, the elements C, O, and Fe coexisted in the sample, which indicated that the composites of NSR20 and Fe_3_O_4_ NPs were successfully fabricated. Meanwhile, the elemental mapping of S and N could be observed in the recovered MNSR1/1 after adsorption, thereby proving that MB was captured by MNSR1/1.

**Fig. 10:**
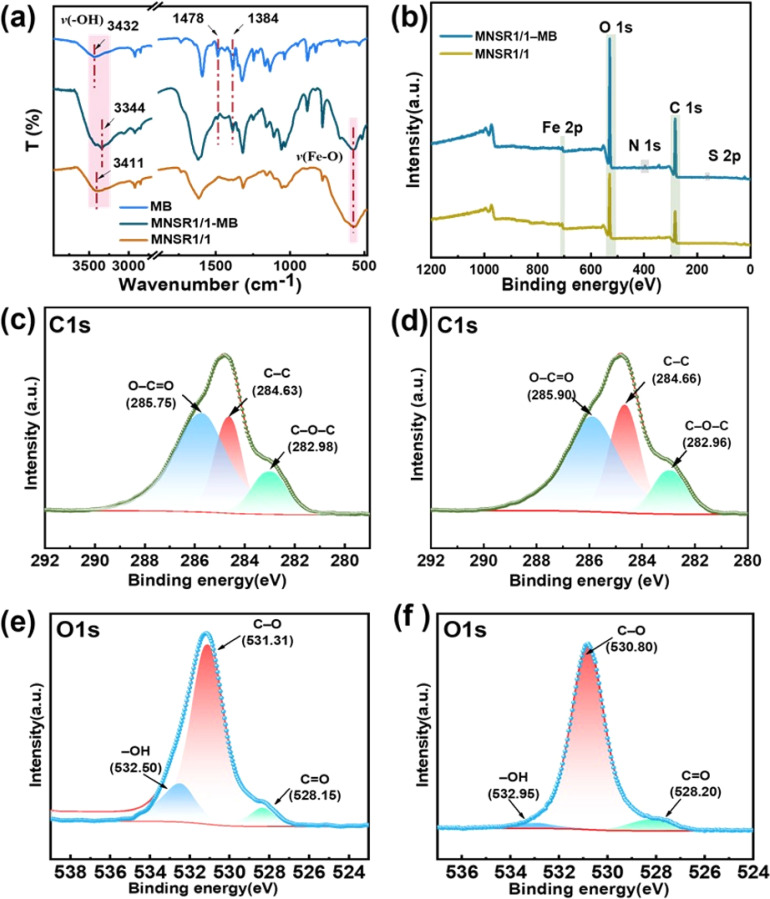
FTIR (a) and XPS (b–f) spectra of the MNSR1/1 samples before and after MB adsorption.

The FTIR spectra of MNSR1/1 before and after adsorption are shown in Fig. 10a. The new absorbance peaks at 1478 and 1384 cm^−1^ were attributed to the C=S^+^ and C=N stretching vibration peaks of MB,^43,44^ respectively. This finding confirmed that MB molecules were bound with MNSR1/1. The peak of the hydroxyl group (–OH) of cellulose in the spectra of MNSR samples before adsorption shifted from 3423 cm^−1^ to 3440 cm^−1^ in the spectra of MNSR1/1 and NSR20 after adsorption (labeled as MNSR1/1-MB and NSR20-MB). This result was possibly due to the hydrogen bonding and electrostatic attraction between MB and –OH groups in MNSR1/1.^45^

The XPS full spectra (Fig. 10b) confirmed the presence of N1s (396.28 eV) and S2p (160.78 eV) as new elemental peaks after adsorption, indicating successful MB adsorption. For the C 1s spectra, MNSR1/1 (Fig. 10c) had three peaks at 285.75, 284.63, and 282.98 eV corresponding to the bonds of O–C=O, C–C, and C–O–C, respectively.^46^ The bonding energy of O–C=O shifted noticeably after adsorption, moving from 285.75 eV to 285.95 eV. This change indicated that an electrostatic interaction occurred between the COO^−^ and MB molecules in the adsorbent.^47^ The peaks of C–C and C–O–C also shifted from 284.63 eV to 284.70 eV and from 282.98 eV to 282.95 eV, respectively, which were influenced by the interaction between MB and MNSR1/1. In the O1s spectra (Fig. 10e and f), after adsorption, the bonding energy of –OH shifted from 532.50 eV to 532.95 eV, and the bonding energy of C=O in the carboxyl groups shifted from 528.15 eV to 528.20 eV. Both peaks were noticeably smaller. This change indicated that oxygen functional groups (–OH and COO^−^) were the main adsorption functional groups. The shift in these groups was also attributed to the electron transfer between MB molecules and the oxygen functional groups (–OH and COO^−^) of MNSR1/1, which was consistent with the results shown in Fig. 10c and d.

**Fig. 11:**
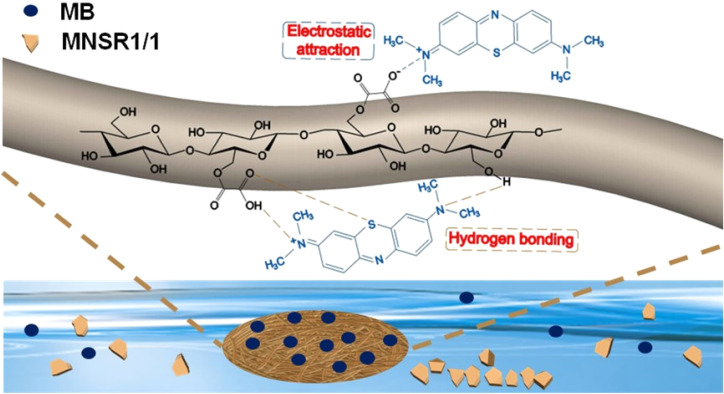
Possible mechanisms of MB adsorption by MNSR1/1.

Therefore, Fig. 11 illustrates the adsorption mechanism and interactions. The adsorption mechanism of MNSR1/1 mainly resulted from the combined action of electrostatic attraction and hydrogen bonding. The oxygen functional groups in MNSR1/1 served as the main active sites for MB adsorption. In particular, the negatively charged oxygen atoms in the carboxyl group contributed to hydrogen bonding and electrostatic interactions between the adsorbent and MB.

## 4. Conclusions

The carboxylated NSR was prepared *via* OA–water pretreatments followed by ultrasonic disintegration. The yield (73.9%) and nanocellulose content (80.2%) of NSR20 were highest among the obtained NSR samples. With increasing OA content from 10% to 60%, the carboxyl content and surface negative charge of NSR samples monotonously increased, thereby improving their affinity. The MNSR adsorbents were prepared with NSR20 and Fe_3_O_4_ NPs *via* a facile self-assembly method. Comprehensive characterization of the MNSR nanocomposite was performed utilizing various techniques, such as FTIR, XRD, SEM, TEM, and XPS. The MNSRs could selectively adsorb the cationic dye of MB from wastewater *via* hydrogen bonding and electrostatic interactions. MNSR1/1 is considered a useful biosorbent for environmental remediation. The adsorption capacity of MNSR1/1 without any modification was so low that it was only employed in the removal of ationic dye from a low dye content of wastewater (*i.e.*, 100 mg L^−1^). In future work, the chemical modification of MNSR will be conducted to improve the adsorption capacity, *i.e.*, amination or carboxylation, because NSR on the surface of MNSR is rich in –OH groups. This work reveals the potential application of MNSR as a dye adsorbent due to its biodegradation, reusability, and low cost.

## Data availability

The data supporting this article have been included as part of the ESI.†

## Author contributions

Xinyi Yang: conceptualization, methodology, software, formal analysis, writing – original draft, investigation; Jingjing Liu: conceptualization, methodology, software, formal analysis; Xuejin Huang: methodology; Hemin Cui: conceptualization; Ligang Wei: writing – review & editing, supervision; Guolin Shao: supervision, investigation; Xu Fu: conceptualization, writing – review & editing, project administration; Na Liu: supervision, investigation; Qingda An: investigation, visualization; Shangru Zhai: investigation, visualization.

## Conflicts of interest

There are no conflicts to declare.

## Supplementary Material

RA-014-D4RA04416A-s001
