# Supplementary material for: Magnetically nanorized seaweed residue for the adsorption of methylene blue in aqueous solutions
Source: RSC Adv. 2024 Jul 29;14(33):23606–20. doi: 10.1039/d4ra04416a (PMC11284532; doi:10.1039/d4ra04416a)
Supplement: RA-014-D4RA04416A-s001 [file RA-014-D4RA04416A-s001.pdf]

Supplementary Information (SI) for RSC Advances.
This journal is © The Royal Society of Chemistry 2024

Electronic Supplementary Information for

Magnetically nanorized seaweed residue for the adsorption of methylene blue in aqueous solutions

Xinyi Yang,[‡a] Jingjing Liu,[‡a] Xuejin Huang,[a] Hemin Cui,[c] Ligang Wei,[*a] Guolin Shao,[a] Xu Fu,[*a,b] Na liu,[a] Qingda An[a,b] and Shangru Zhai[a,b]

[a] *School of Light Industry and Chemical Engineering, Dalian Polytechnic University, Dalian 116034, China*

[b] *Liaoning Key Lab of Lignocellulose Chemistry and BioMaterials, Liaoning Collaborative Innovation Center for Lignocellulosic Biorefinery, Dalian Polytechnic University, Dalian Polytechnic University, Dalian 116034, China*

[c] *Dalian Zhonghuida Scientific Instrument Co. LTD., Dalian 116023, China*

[*] Corresponding author. Tel: +86 0411 86323726. Email address: weilg@dlpu.edu.cn (L. Wei); fuxumail@163.com (X. Fu).

[‡] These authors contributed equally to this paper.

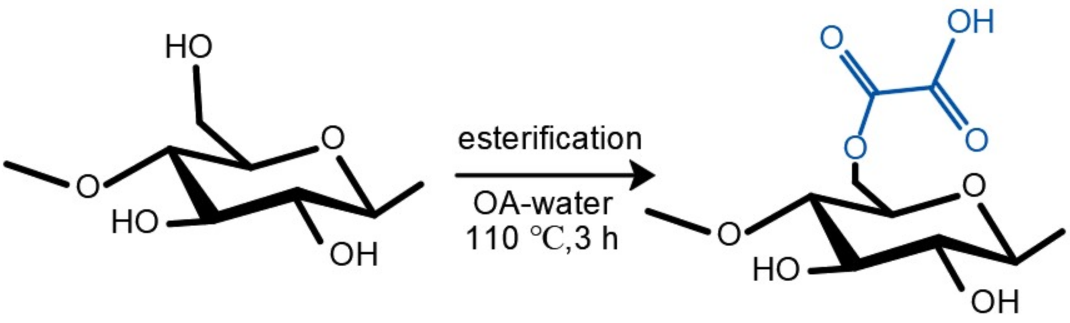

Scheme S1 Carboxylic functions of SR cellulose in the OA-water pretreatment.

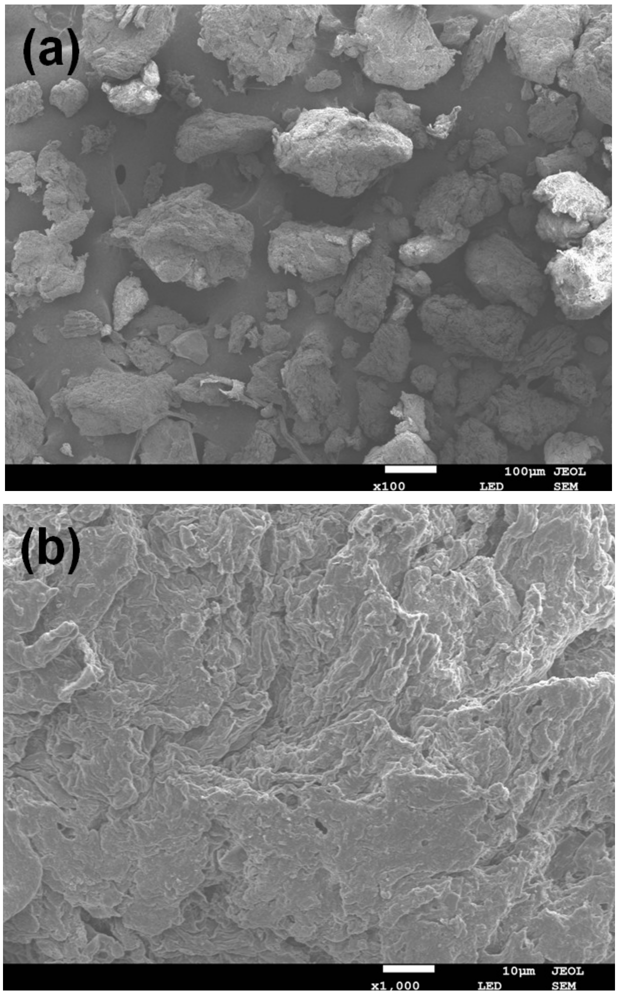

Fig. S1 SEM images of SR.

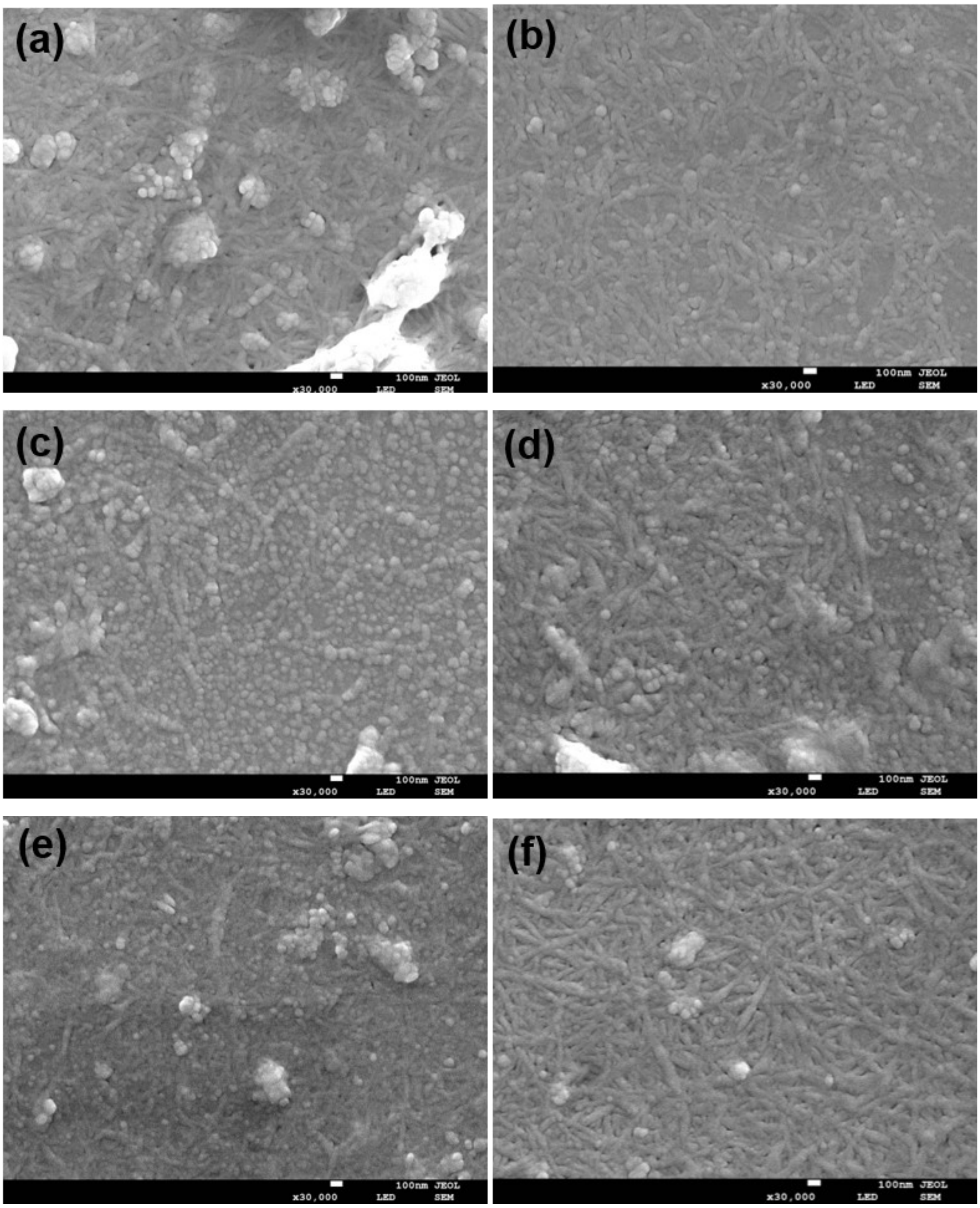

Fig. S2 SEM images of prepared NSR samples. NSR10 (a), NSR20(b), NSR30(c), NSR40(d), NSR50(e), NSR60(f).

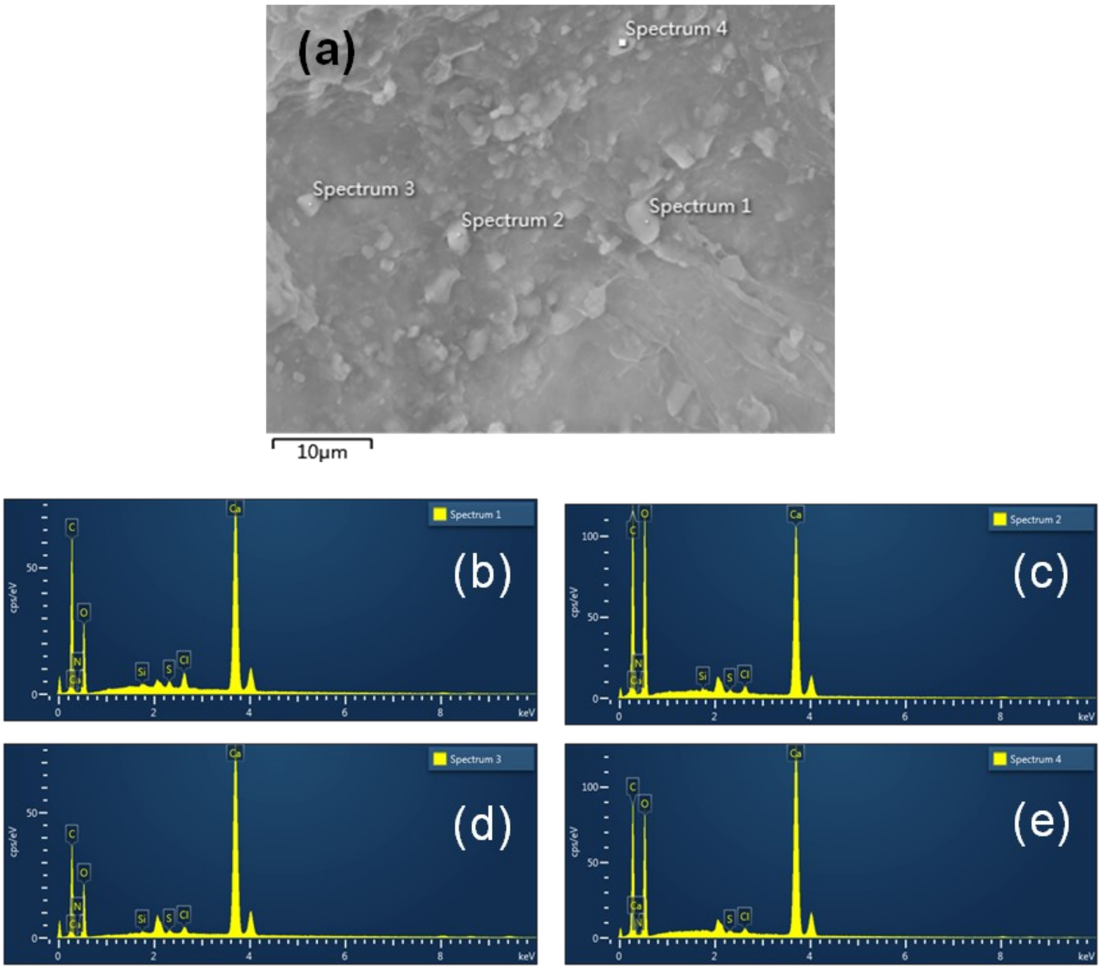

Fig. S3 SEM images of NSR samples (a) and EDS spectra (b-e)

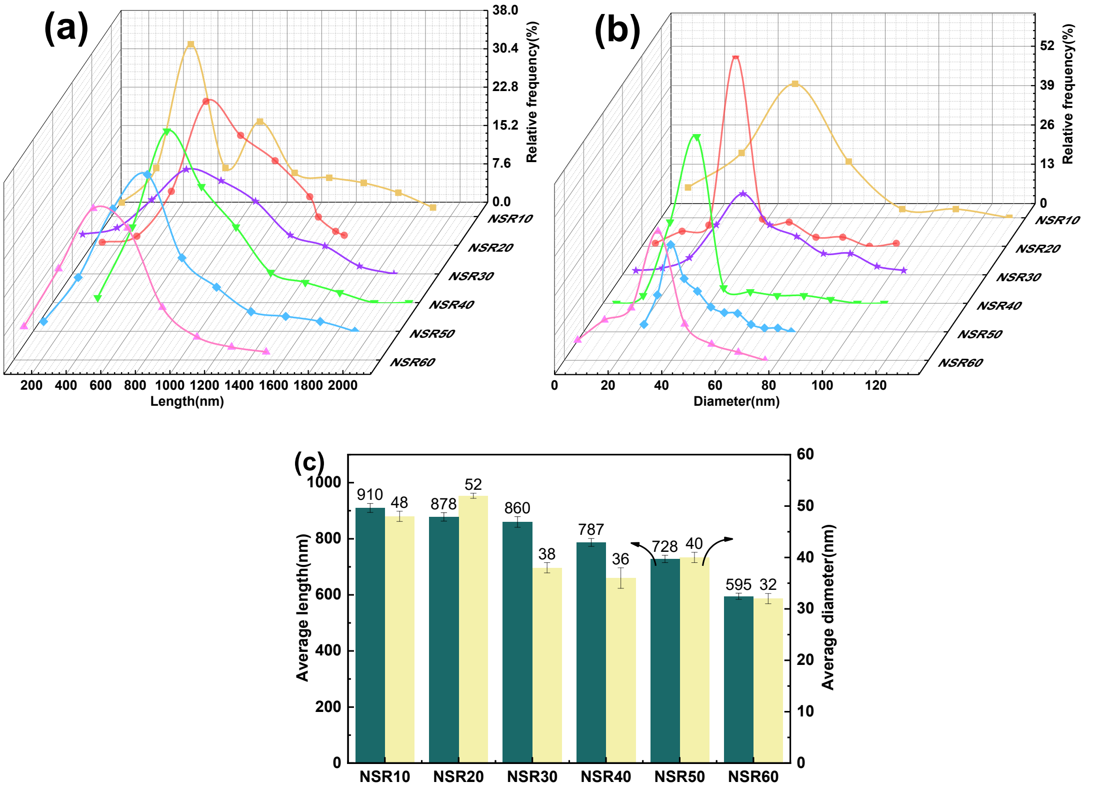

Fig. S4 Size distributions of the prepared NSR-NC samples: length (a); diameter (b); and average length and diameter (c).

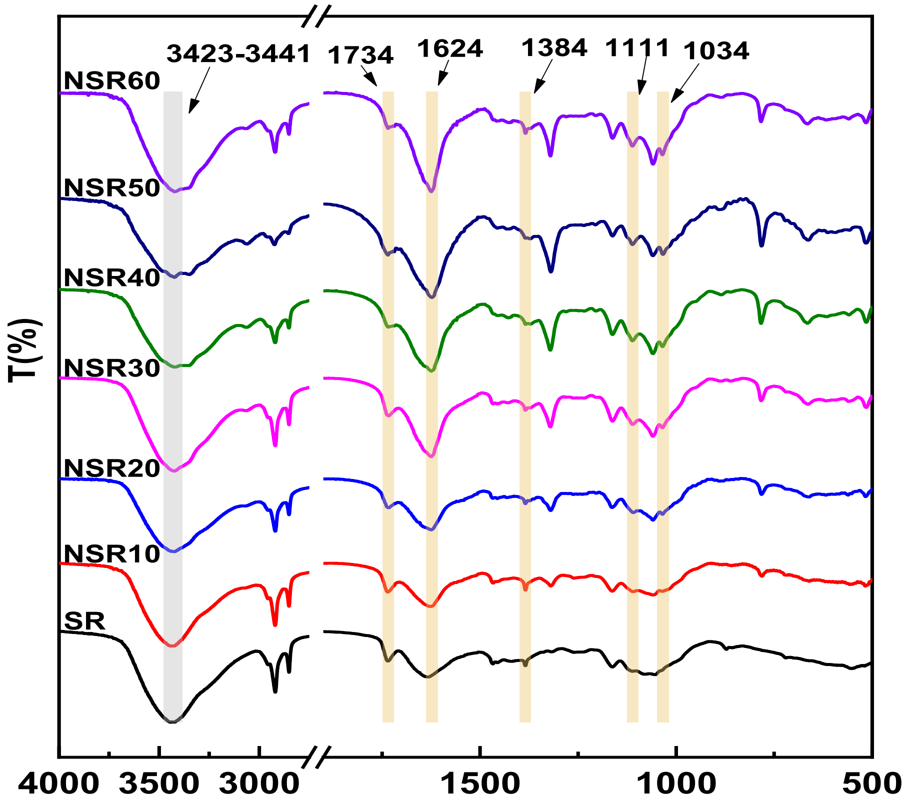

Fig. S5 FTIR spectra of SR and prepared NSRs.

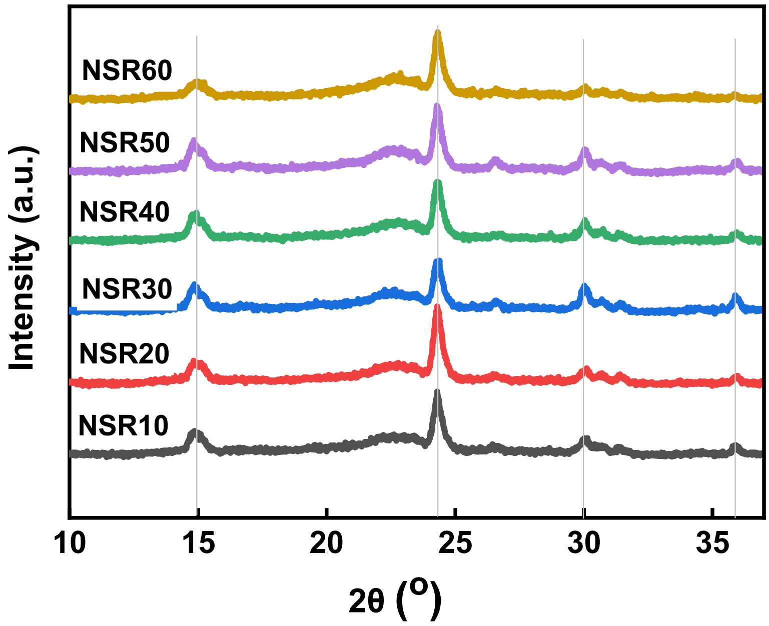

Fig. S6 XRD patterns of prepared NSRs.

Table S1 Properties of SR and prepared NSRs.

sample	S_{BET} (m^2/g)	carboxyl group content (mmol/g)
SR	36.91	1.79±0.04
NSR10	57.60	4.58±0. 13
NSR20	48.89	4.74±0.21
NSR30	43.54	4.96±0.19
NSR40	62.03	6.01±0.26
NSR50	58.84	6.26±0.29
NSR60	57.38	6.73±0.38

Table S2 Residual rate of Fe_3O_4 NPs during preparing magnetic composites and density of surface charge of used NC materials.

sample	residual rate of Fe_3O_4 NPs(%)	density of surface charge (μeq/g)
NC-1	18.9±0.7	-13±2
NC-2	46.3±2.4	-42 ±5
NSR-SA	8.5±0.5	-87±3
NSR20	1.4±0.2	-350±12

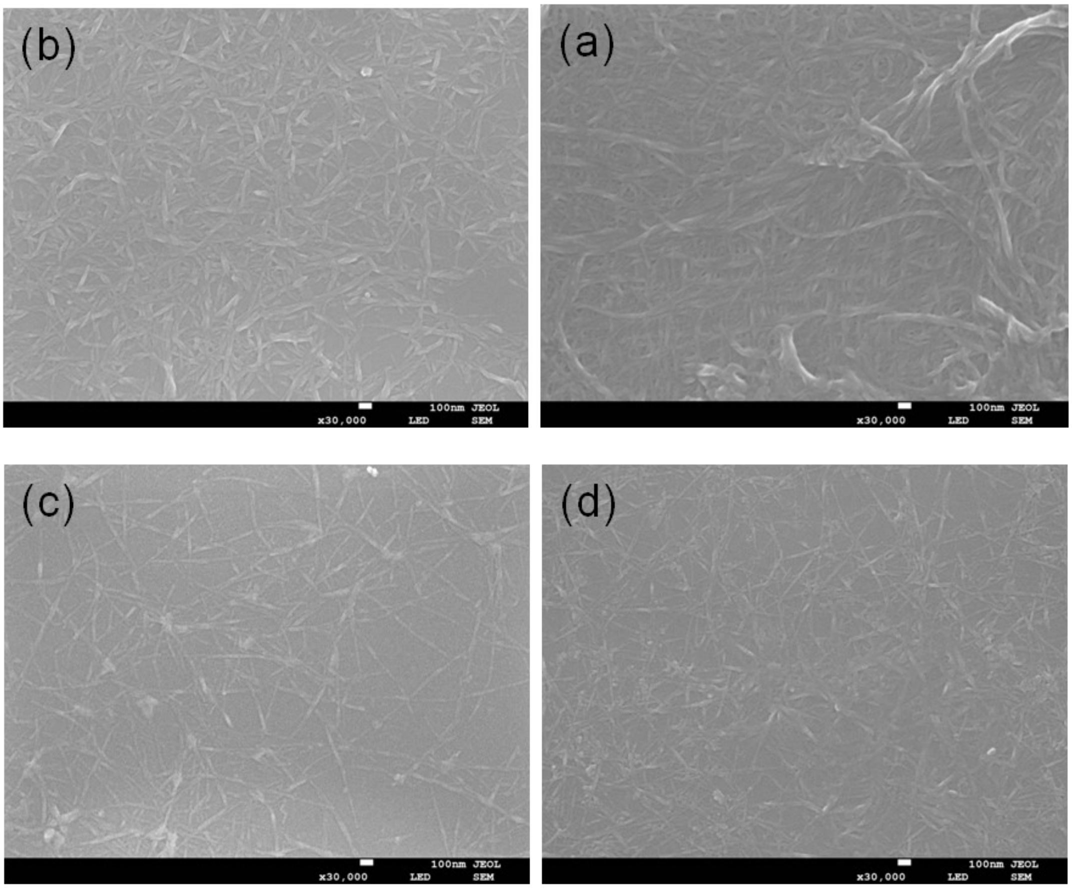

Fig. S7 SEM images of NC-1(a), NC-2(b), NSR-SA(c), and NSR20 (d).

Table S3 Comparison of MNSR1/1 with other adsorbents for MB dye.

Adsorbents	Recycle times/RE(%)	q_{max}(mg/g)	Ref
Ni $(OH)_2$/polyacrylamide composite	5/90.91	14.3	[S1]
cationic poly (styrene sulfonate)-incorporated anionic chitosan beads	3/47.20	42.21	[S2]
Bacterial cellulose and expanded vermiculite composite	-/-	92.16	[S3]
cellulose/polyvinyl alcohol /expanded graphite 3D porous foam	5/71.93	110.81	[S4]
UiO-66-NH_2 functionalized with an ionic polymer poly (2-acrylamido-2-methylpropane sulfonic acid)	-/-	120.34	[S5]
polyaniline/zeolite-A composite	-/-	270.9	[S6]
The activated carbons obtained by chemical activation with potassium carbonate	-/-	296	[S7]
MNSR1/1	5/82.71	184.25	this work

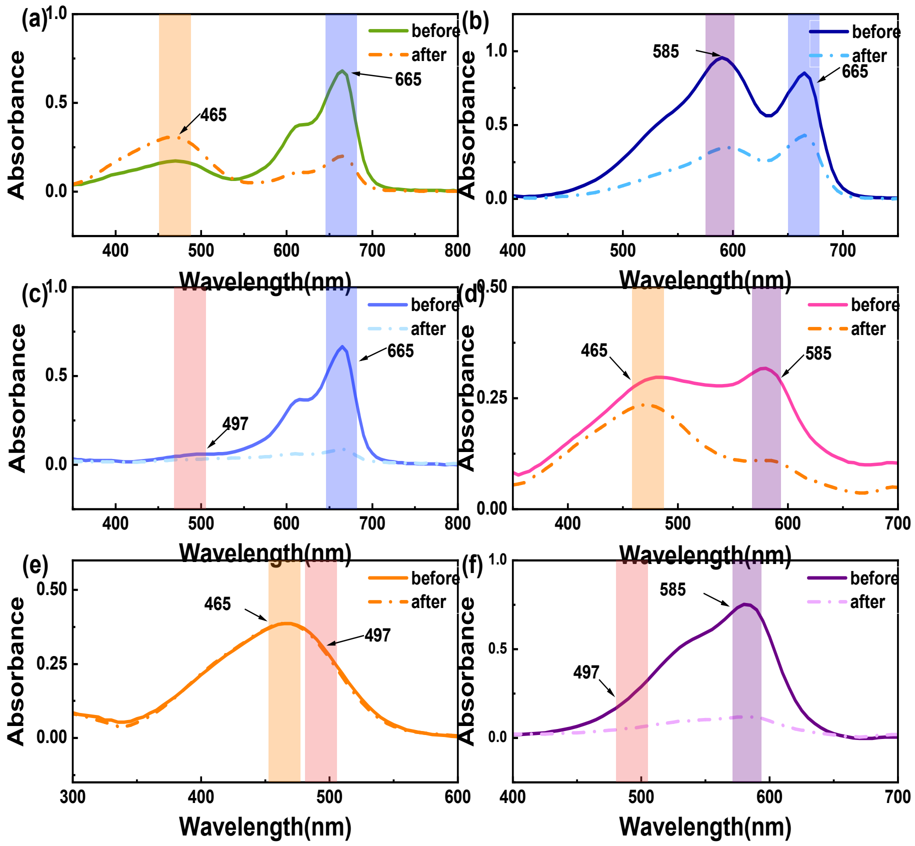

Fig. S8 The UV full-spectra of MB+MO (a), MB+MV (b), MB+CR (c), MO+MV (d), MO+CR (e) and MV+CR (f) dyes mixture in the selective adsorption of MNSR1/1.

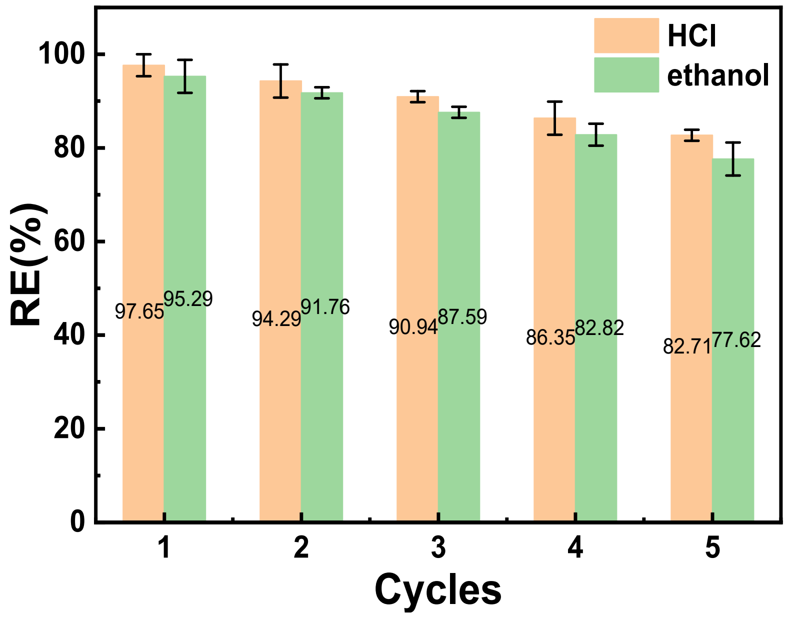

Fig. S9 Recycled experiments of MNSR1/1.

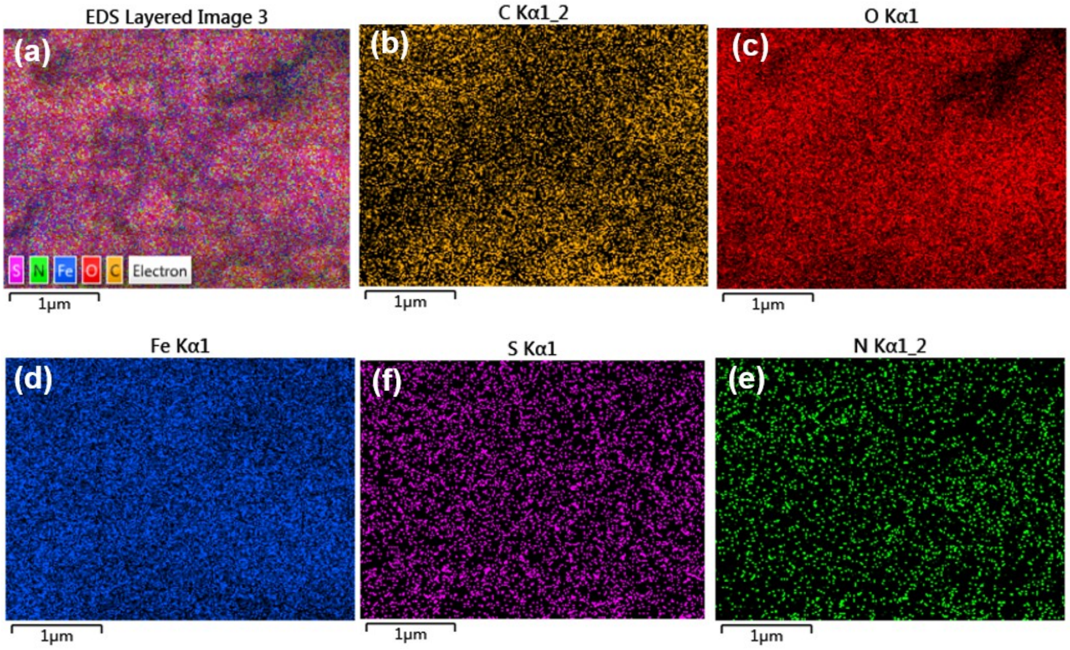

Fig. S10 Elemental mappings of layered image (a), C (b), O (c), N(d), Fe(e), S (f), N (e) in MNSR1/1.

References.

S1. A. Marey, M. Adel, A. M. A. E. Naggar, A. A. El-Zahhar and M. H. T. Taha, *Dalton Trans.*, 2023, **52**, 14194-14209.

S2. K. S. Lau, N. A. S. Azmi, S. X. Chin, S. Zakaria and C. H. Chia. *Polymers*, 2023, **15**, 1269.

S3. X. Bai, Z. Liu, P. Liu, Y. Zhang, L. Hu and T. Su, *Polymers*, 2023, **15**, 2342.

S4. X. Tan, Q. Peng, T. Subrova, J. Saskova, J. Wiener, M. Venkataraman, J. Militky, W. Xiong, J. Xu A. R. Mahendran and H. Lammer, *J. Nat. Fibers*, 2023, **20**, 2190189.

S5. H. Fan, Z. Zhang, Y. Chen, Z. Wang, H. Wen, S. Tian, S. Chen, H. Zhao, Y. He and Y. Wang, *Dalton Trans.*, 2023, **52**, 5028-5033.

S6. A. T. Zaidalkilani, A. M. Farhan, I. R. Sayed, A. M. El-Sherbeeny, W. A. Zoubi, A. Al-Farga and M. R. Abukhadra. *Molecules*, 2023, **28**, 7168.

S7. D. Paluch, A. Bazan-Wozniak, A. Nosal-Wiercińska and R. Pietrzak. *Molecules*, 2023, **28**, 6306.
